# Implementation of theoretical non-photochemical quenching (NPQ_(T)_) to investigate NPQ of chickpea under drought stress with High-throughput Phenotyping

**DOI:** 10.1038/s41598-024-63372-6

**Published:** 2024-06-17

**Authors:** Madita Lauterberg, Henning Tschiersch, Yusheng Zhao, Markus Kuhlmann, Ingo Mücke, Roberto Papa, Elena Bitocchi, Kerstin Neumann

**Affiliations:** 1 Leibniz Institute of Plant Genetics and Crop Plant Research (IPK), Seeland, Germany; 2 Marche Polytechnic University (UNIVPM), Ancona, Italy

**Keywords:** Chickpea, Chlorophyll fluorescence, Non-photochemical quenching, High-throughput Phenotyping, Drought stress, Plant breeding, Natural variation in plants, Non-photochemical quenching, Drought, Agricultural genetics

## Abstract

Non-photochemical quenching (NPQ) is a protective mechanism for dissipating excess energy generated during photosynthesis in the form of heat. The accelerated relaxation of the NPQ in fluctuating light can lead to an increase in the yield and dry matter productivity of crops. Since the measurement of NPQ is time-consuming and requires specific light conditions, theoretical NPQ (NPQ_(T)_) was introduced for rapid estimation, which could be suitable for High-throughput Phenotyping. We investigated the potential of NPQ_(T)_ to be used for testing plant genetic resources of chickpea under drought stress with non-invasive High-throughput Phenotyping complemented with yield traits. Besides a high correlation between the hundred-seed-weight and the Estimated Biovolume, significant differences were observed between the two types of chickpea *desi* and *kabuli* for Estimated Biovolume and NPQ_(T)_. *Desi* was able to maintain the Estimated Biovolume significantly better under drought stress. One reason could be the effective dissipation of excess excitation energy in photosystem II, which can be efficiently measured as NPQ_(T)_. Screening of plant genetic resources for photosynthetic performance could take pre-breeding to a higher level and can be implemented in a variety of studies, such as here with drought stress or under fluctuating light in a High-throughput Phenotyping manner using NPQ_(T)_.

## Introduction

Chickpea (*Cicer arietinum* L.) is a protein-rich legume that is becoming increasingly popular due to its health benefits and as replacement for energy intensive production of animal-based protein. Drought stress is reported to reduce chickpea yields by about 40–50% worldwide^1,2^. This is of the utmost importance, as drought events will occur more frequently in Europe in the future^3^. A good strategy is to improve yield potential and stability in challenging conditions by incorporating plant genetic resources into pre-breeding and modern breeding programs and exploiting their diversity to improve drought tolerance in chickpea^4,5^. Cultivated chickpeas are grouped into two types, based on their origin and their use in agriculture: *desi* and *kabuli*^6^. While the multi-colored *desi* is mainly used and cultivated in Indian subcontinent, the lighter-colored *kabuli* is mainly grown in the Mediterranean Basin. In pot and field studies, *desi* has been described as more tolerant to drought stress than *kabuli*, thus *desi* plant genetic resources are expected to be more promising for the identification of drought tolerant genotypes^7–10^. The assessment of photosynthetic capacity might be an interesting trait to be introduced for selecting genotypes to be included in breeding programs aimed to develop more resilient and drought tolerant plants.

Over the last decade, High-throughput Phenotyping (HTP) has developed into a state-of-the-art method. Plant genetic resources of crops such as barley (*Hordeum vulgare* L.), maize (*Zea mays* L.), pea (*Pisum sativum* L.) and chickpea, have been analysed on several HTP systems and tested for drought or cold tolerance using estimated biovolume for shoot biomass as the main trait^11–14^. The combination of morphological and physiological traits, measured with different imaging systems such as red–green–blue (RGB) or chlorophyll fluorescence, can be used to deeply characterize plant genetic resources and to investigate their genetic architecture in a spatio-temporal pattern.

When light energy is captured by chlorophyll, it is passed to photochemistry, non-photochemical quenching (NPQ) or fluorescence to avoid production of reactive oxygen species and photodamage^15^. The chlorophyll fluorescence of photosystem II accounts for 0.6 to 3% of the absorbed light and can be measured with pulse-amplitude modulation (PAM)^16^. The operating efficiency of photosystem II (ФPSII, F_q_′/F_m_′), measured on fully light-adapted plants, and the maximum quantum yield (F_v_/F_m_), measured on fully dark-adapted plants, have been measured frequently with different growth conditions and for many species such as brassica, rice, maize or tobacco (*Nicotina tabacum*)^14,17,18^. The advantage of these two traits F_v_/F_m_ and ФPSII is that the measurement is fast and therefore feasible for HTP.

NPQ is a mechanism to protect plants from photodamage and is described by two main components: fast relaxation (qE) and slow relaxation (qI)^15^. Xanthophylls interconvert between zeaxanthin and violaxanthin in a pH dependent manner, which modulates the qE component of NPQ. A conformational change of the PS-II unit and the PsbS protein is achieved by the binding of protons and xanthophylls to specific sites of the antenna complexes. qE is rapidly reversible and qI is a measure of slowly relaxing quenching, the main mechanism of which is photoinhibition.

Under fluctuating light conditions, a rapid conformational change in the antenna complexes is beneficial, implying a dynamic NPQ. Modelling has shown that the losses of potential carbon gain are between approximately 13 and 30% and strongly depend on the dynamically relaxation kinetics of the NPQ^19^. Studying photosynthesis in fluctuating light simulates conditions that are as close as possible to the outside environment where light conditions are often not constant, so the photosynthesis has to adapt to maximize daily carbon gain^20^. In experiments with tobacco (*Nicotiana tabacum*) and soybean (*Glycine max* L.), it was discovered that the ability to adapt dynamically with NPQ when changing from high to low light led to a 33% increase in yield and a 15% increase in dry matter productivity^21,22^.

NPQ is calculated from F_m_ and F_m_′ which means fully light- and fully dark-adaptation are required^23^. Full light adaptation takes a minimum of 30 min and a dark adaptation a minimum of 1 h^24^, thus the measurement is very time consuming and therefore not feasible for HTP^25^.

Tietz et al.^26^ presented a protocol for measuring theoretical NPQ (NPQ_(T)_) in *Arabidopsis thaliana* that was not only faster but also worked under light-adapted conditions. To measure NPQ_(T)_, F_v_/F_m_ was not measured but a fixed value was used, taking advantage of the fact, that F_v_/F_m_ is a consistent value within a species^24^. After a measurement of F_q_′/F_m_′, far-red light was applied. Far-red light ensures that photosystem II (PSII) is fully opened and that the first stable electron acceptor of PSII the plastoquinone A (Q_A_) and the plastoquinone pool is oxidized^27^. This avoids the waiting time in which PSII is oxidized and the final F_0_′ determination can be carried out more quickly. NPQ_(T)_ has already been implemented in carry-on measurement tools and tested for species such as maize and soybean^28–30^. NPQ_(T)_ appears to be suitable for High-throughput Phenotyping and could help to exploit plant genetic resources for future photosynthesis improvement.

## Objective

In the present study

1. A protocol for quantification of NPQ_(T)_ was implemented which serves as proxy for NPQ;
2. The trait NPQ_(T)_ was validated as suitable method for High-throughput Phenotyping (HTP);
3. NPQ_(T)_ was compared with other image-based traits and yield traits and used as an example for screening plant genetic resources of chickpea under control and drought stress conditions.

## Results

The results of the HTP experiment with 60 chickpea genotypes and 2 replicates each in control and drought stress treatment, are described below. During the plant establishment phase with control treatment for all plants, the NPQ_(T)_ method was validated. For this purpose, F_v_/F_m_, NPQ and NPQ_(T)_ were measured. Then, the drought stress was initiated and the NPQ_(T)_ method was implemented during the period of drought stress and in the following recovery phase, and finally, yield traits were determined at maturity.

### Description of data: data availability, repeatability and capacity of measurements

For method-validation of NPQ_(T)_, all 240 plants were measured for F_v_/F_m_ in the control treatment on the night of day 4 to 5 after the transfer (DAT) of the pots with the plants from the pre-cultivation in the greenhouse to the HTP system. Furthermore, 23 plants corresponding to 20 different genotypes were measured for NPQ and NPQ_(T)_ in control treatment during the night of DAT 5 to 6 (Table S1).

For the method-implementation NPQ_(T)_ during the drought stress and recovery from DAT 8–37, the raw data of image-derived traits of daily HTP has been inspected. The missing rate for Estimated Biovolume and Mean Color Value for the five DAT 12, 18, 19, 24 and 35 was higher than 20% (Table S2). For the red to green color ratio and plant height, the three DAT 19, 24 and 35 had a missing rate higher than 20%. It should be noted that the incomplete DAT were not the key DAT on which chlorophyll fluorescence measurements were conducted or drought stress was maximal or recovery were induced. Moreover, the rate of missing raw data from individual replicates was not higher than 6.6% across all DAT (Table S3). The missing rate for ФPSII and NPQ_(T)_ was on average 12.9% across the four DATs (Table S4).

The repeatability, which is an indicator of the consistency of the repeated measurement and varies between 0 and 1, with 1 representing the highest possible consistency, was calculated. For the method-validation, a high repeatability of 0.8 has been observed for F_v_/F_m_. For NPQ the repeatability was 0.69 and for NPQ_(T)_ it was 0.48.

For the method-implementation, a high repeatability of more than 0.8 was calculated for Estimated Biovolume for the control treatment and was marginally lower for the drought stress with 0.7 (Figure S1). Repeatabilities of 0.8 on average also result for plant height and Mean Color Value for the two treatments (Figure S2). For plant height, the repeatability for both treatments increased with increasing experiment duration. The repeatability was at an average value of 0.65 for red to green color ratio. Furthermore, red to green color ratio had more fluctuations in repeatability across the time course.

In the control treatment the repeatability for ФPSII was on average 0.66 and for NPQ_(T)_ 0.47. Furthermore, in the drought stress treatment, the repeatability for ФPSII and NPQ_(T)_ was on average 0.4 (Figure S3).

For the yield trait, the repeatability was highest for the weight of seeds with 0.66 for control and 0.57 for drought stress treatment and lowest for number of empty pods with 0.14 for control and 0.3 for drought stress treatment (Figure S4). For the hundred-seed-weight, the repeatability was 0.45 for control and 0.27 for drought stress treatment.

For the method-validation, in the night from DAT 5 to 6, 9.4 dark-adapted plants per hour have been measured for NPQ. The average capacity for NPQ_(T)_ measurements for the method-implementation and across the four measurement DATs 38.8 fully light-adapted plants per hour with a maximum of 42 plants per hour. This numbers for the capacity included the rotation of the conveyer belts, which transport the carriers with each one plant to the imaging chamber.

### Method-validation of NPQ_(T)_ as proxy for NPQ

For all 60 genotypes, the F_v_/F_m_ average was 0.856 ± 0.002 (Table S5). Among the 23 measurements of replicates for NPQ the average was 0.685 ± 0.084 and for NPQ_(T)_ it was 1.826 ± 0.147 (Table S6).

**Figure 1:**
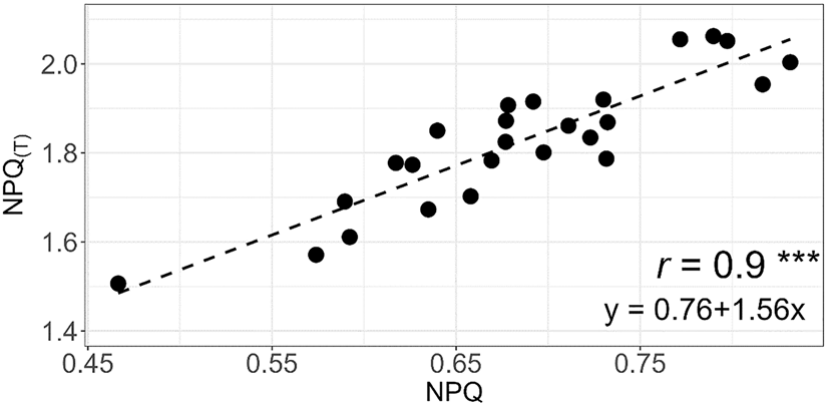
Correlation of NPQ and NPQ_(T)_. *r* = coefficient of correlation. ***p < 0.001. Based on raw data of 20 genotypes and 23 replicates.

The correlation between NPQ and NPQ_(T)_ could be explained by a regression with a coefficient of correlation *r* of 0.9 (Fig. 1).

### Impact of drought stress on HTP image-derived and chlorophyll fluorescence traits

Estimated Biovolume was used as proxy for the shoot biomass and reduced accumulation of biomass can be used as indication for the impact of drought stress.

**Figure 2:**
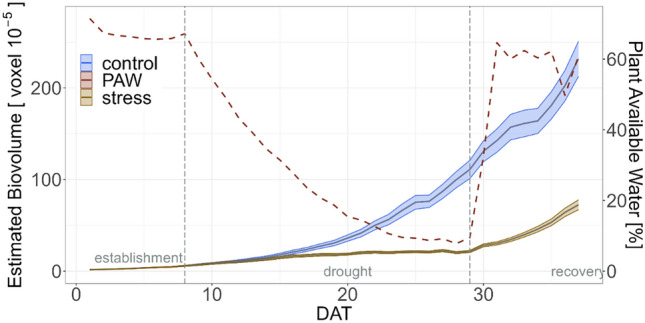
Estimated Biovolume in drought stress and control treatments. The red dashed line indicates the plant available water to which the secondary axis refers to. The two vertical grey dashed lines indicate the different phases of the experiment: establishment, drought and recovery. The shadows describe the 95% confidence interval; as long as the shadows of the individual lines do not overlap, the significance level of *α* = 0.05 was reached. Based on average of BLUEs within the experiment of all 60 genotypes. Interpolated on DAT 12, 18, 19, 24, 35. DAT = days after transferring to the High-throughput Phenotyping system.

From DAT 17 onwards, at an average plant available water content of 23%, the Estimated Biovolume of the chickpea plants of drought stress and control treatments differed significantly (Fig. 2, Table S7). On DAT 28, at the last day of drought stress, average of Estimated Biovolume for the control plants was 99.1 ± 35 [voxel 10^−5^] while for drought stress it was 20.3 ± 4.5 [voxel 10^−5^] (Table S8). This was a reduction of 79.5% in drought stress compared to the control treatment. On DAT 37, after application of drought stress and a recovery phase, the average of Estimated Biovolume for the plants in control treatment was 231.5 ± 75.4 [voxel 10^−5^] and for drought stress it was 72.5 ± 20.9 [voxel 10^−5^], describing a difference of Estimated Biovolume for plants in drought stress of 68.7% compared to those in the control treatment.

A significant effect of drought stress on plant height, meaning smaller chickpea plants in drought stress, was observed on DAT 19 at a plant available water content of 18.7% (Figure S5, Tables S7, S8). On DAT 28, the difference of drought stress compared to the control treatment was 29% (control = 515 ± 72 [mm]; stress = 365 ± 57[mm]) and at the end of the experiment on DAT 37 the difference was 26.9% between the two treatments. From DAT 16 until DAT 36, starting at a plant available water content of 27%, the chickpeas of the drought stress and control treatments differed significantly for Mean Color Value. On DAT 28, the last day of drought stress, the average of Mean Color Value for the control replicates was 0.278 ± 0.004 [hue] and for drought stress it was 0.296 ± 0.005 [hue] (Table S8). This was a difference of − 6.6% in drought stress compared to the control treatment and gave the impression of a darker green in drought stress treatment. In addition, the red to green color ratio was higher in drought stress treatment than in control treatment on DAT 28 (control = 0.125 ± 0.04; stress = 0.22 ± 0.06).

**Figure 3:**
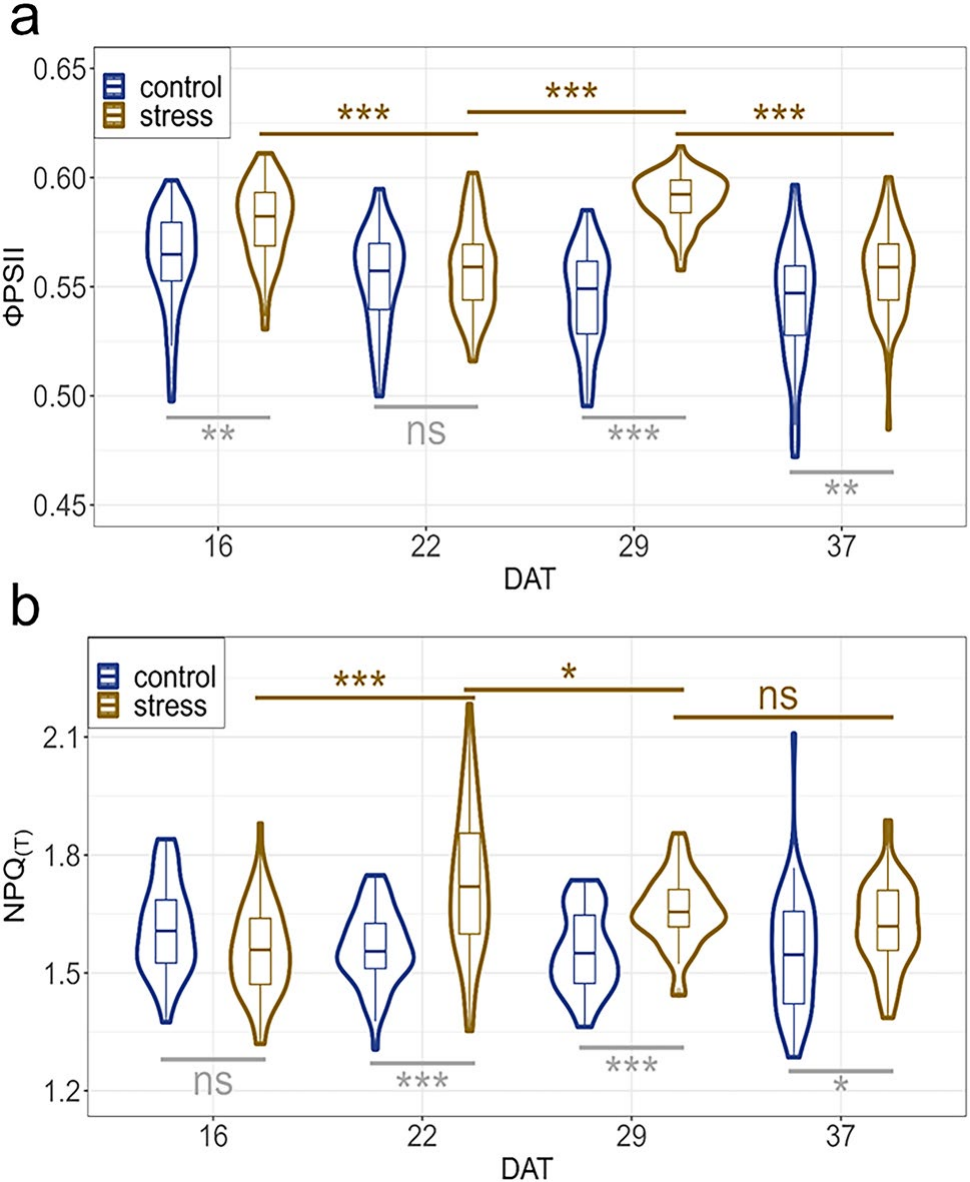
Chlorophyll fluorescence traits under drought stress and recovery. (**a**) ФPSII; (**b**) NPQ_(T)_. DAT 16 = 8 days of drought stress; DAT 22 = 14 days of drought stress; DAT 29 = first day of recovery; DAT 37 = 8 days of recovery. Based on BLUEs within the experiment. DAT = days after transferring to the High-throughput Phenotyping system. The brown lines above the plots refer to the significance between the adjacent DAT within the drought stress treatment. The gray lines below the plots refer to the significance between the two treatments on each DAT. All significances can be found in Table S12. ns = not significant; ***p < 0.001; **p < 0.01; *p < 0.05.

For ФPSII the average across all phenotypes analyzed in control treatment ranged from 0.54 ± 0.02 to 0.56 ± 0.02 for the four measured DATs (Fig. 3, Tables S11, S12). In drought stress ФPSII was between 0.56 ± 0.02 and 0.59 ± 0.01.

The two treatments drought stress and control differed significantly on DAT 16, 29 and 37 and on all three DATs the drought stressed chickpea plants had a slightly higher ФPSII.

The average of NPQ_(T)_ in the control treatment varied between 1.55 ± 0.15 and 1.61 ± 0.11 for the four DATs and there were no significant differences (Fig. 3, Tables S11, S12).

The plants in drought stress had a significantly different NPQ_(T)_ between DAT 16 and 22 and between DAT 22 and 29 with the averages of 1.56 ± 0.11, 1.73 ± 0.18, 1.66 ± 0.09 and 1.62 ± 0.11 at the respective DATs.

On DAT 22, 29 and 37, the plants differed significantly from each other between the two treatments. At all three DATs, plants in drought stress had a higher value for NPQ_(T)_, with the difference being highest at DAT 22.

There was a significant negative effect of drought stress on the yield traits (Figure S6, Tables S9, S10). The hundred-seed-weight showed a reduction of 58%, in drought stress compared to the control treatment with an average of 200.2 ± 100.2 g in control to 82.2 ± 73.4 in drought stress. For the other traits, the difference of drought stress compared to the control treatment varied between 49.2% for number of empty pods and 77.5% for weight of seeds (Table S9). For all traits the difference was significant (Table S10).

### Interaction of traits

When comparing NPQ_(T)_ and ФPSII under the control treatment, NPQ_(T)_ had a higher coefficient of variation among all DATs and for both treatments (Figure S7). This was pronounced on DAT 22, when the coefficient of variation for drought stress was 10.5 for NPQ_(T)_ and 3.6 for ФPSII.

There were high correlations of* r* = 0.61 to 0.7 between Estimated Biovolume and hundred-seed-weight for DATs 16, 22, 29 and 37 for both treatments combined (Table S13). Similarly, there were correlations of *r* = 0.42 to 0.62 between Estimated Biovolume and weight of seeds for these DATs and both treatments combined. For both treatments combined, there was a significant negative correlation of *r* =  − 0.19 to − 0.26 between NPQ_(T)_ and hundred-seed-weight for DATs 22, 29 and 37. There was also a negative correlation of *r* =  − 0.34 to − 0.45 between NPQ_(T)_ and Estimated Biovolume for both treatments combined for DAT 22, 29, 37.

On DAT 22, after 14 days of drought stress, NPQ_(T)_ and ФPSII were significantly positively correlated in the control treatment (*r *= 0.44) and significantly negatively correlated in the drought stress treatment (*r* =  − 0.58) (Table S13).

The correlation between NPQ_(T)_ and Estimated Biovolume was not significant on DAT 22 in control treatment (Fig. 5, Table S13). For drought stress, the correlation was significant and amounted to *r* = 0.53 and for the difference of drought stress compared to the control treatment, the significant correlation amounted to *r* =  − 0.34.

### The plasticity for NPQ_(T)_ can differentiate *desi* and *kabuli* chickpeas

On DAT 22, *desi* had significantly less Estimated Biovolume than *kabuli* under control treatment (*desi* = 43.57 ± 14.88; *kabuli* = 56.64 ± 19.91 (Fig. 5, Tables S14, S15). For drought stress, Estimated Biovolume was similar between both types of chickpea (*desi* = 20.1 ± 4.29; *kabuli* = 21.6 ± 4.97). Thus, *kabuli* had a significantly larger difference of Estimated Biovolume under drought stress compared to control treatment, than *desi*.

**Figure 4:**
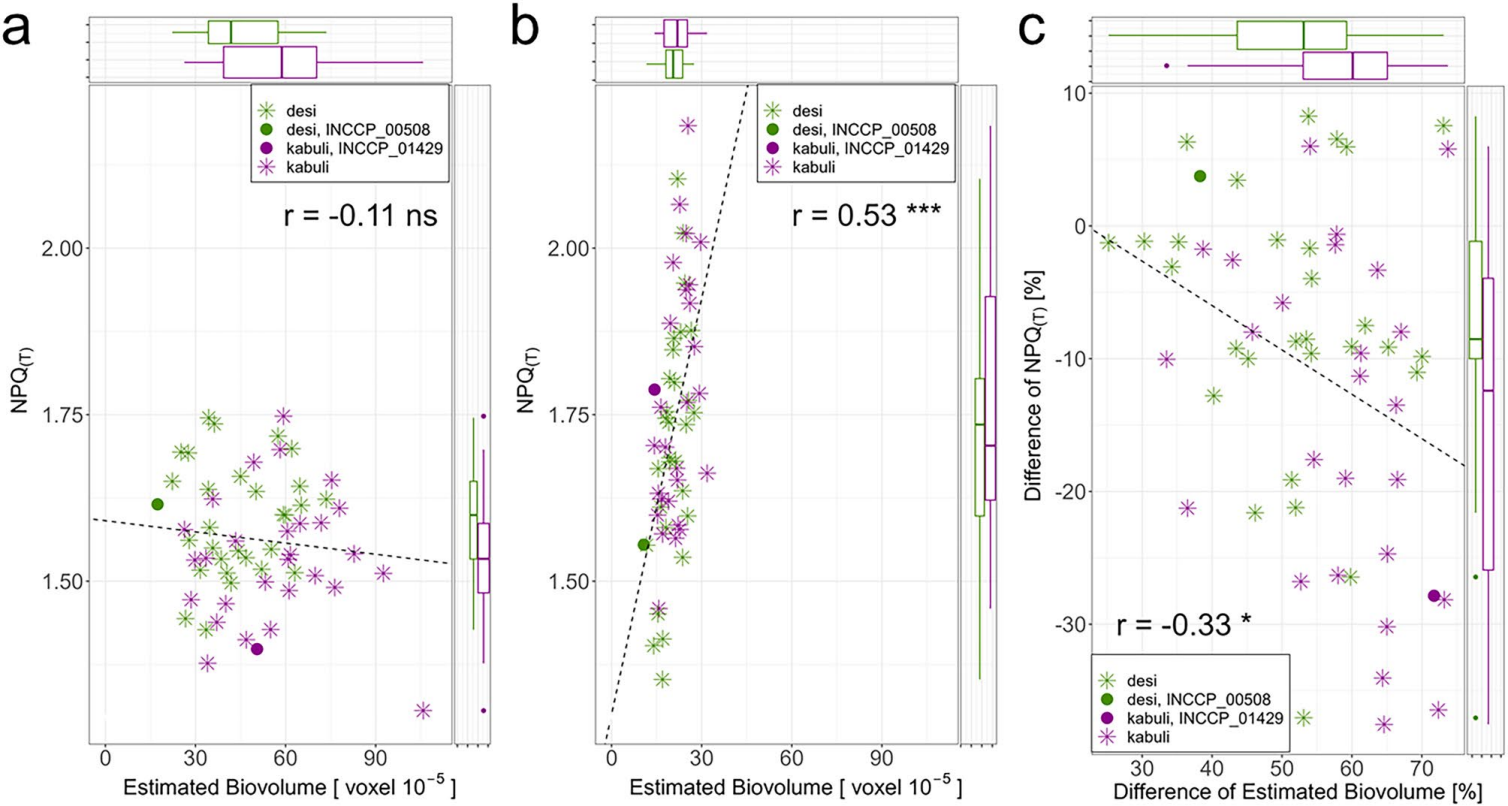
Correlations between Estimated Biovolume and NPQ_(T)_ on DAT 22 for *desi* and *kabuli*. (**a**) for control treatment; (**b**) for drought stress treatment; (**c**) for the difference of drought stress compared to control treatment. Side-boxplots show the distribution of *desi* and *kabuli*, with the 25% and 75% quantiles and the median described by the boxes and for the corresponding axis. Based on Blues within experiment. Difference [%] = (1 − (drought stress/control)) * 100. *r *= coefficient of correlation; ns = not significant; *p < 0.05; ***p < 0.001. DAT 22 = days after transferring chickpeas to the High-throughput Phenotyping system with 14 days of drought stress. The two chickpea types, *desi* and *kabuli*, and one exemplary and representative genotype of each type were highlighted.

On DAT 22 the NPQ_(T)_ of *desi* and *kabuli* differed in control treatment significantly (*desi* = 1.59 ± 0.08; *kabuli* = 1.53 ± 0.1), but not in drought stress treatment (*desi* = 1.7 ± 0.18; *kabuli* = 1.76 ± 0.18). Therefore, *desi* had a significantly lower difference of drought stress compared to the control treatment of NPQ_(T)_ than *kabuli* (Fig. 4, Tables S14, S15).

An exemplary and representative genotype was highlighted for each type of chickpea. For the *desi* genotype INCCP_00508 (*desi*, breeding material, Middle East), the NPQ_(T)_ was 1.62 in the control treatment and 1.55 in drought stress, with a small difference of drought stress compared to the control treatment for Estimated Biovolume of 38% (control = 17.35; drought stress = 10.71). For the *kabuli* genotype INCCP_01429 (*kabuli*, landrace, Middle East), NPQ_(T)_ was 1.4 in the control treatment and significantly higher at 1.79 in drought stress, and there was a high difference of Estimated Biovolume of 71% (control = 50.49; drought stress = 14.27).

**Figure 5:**
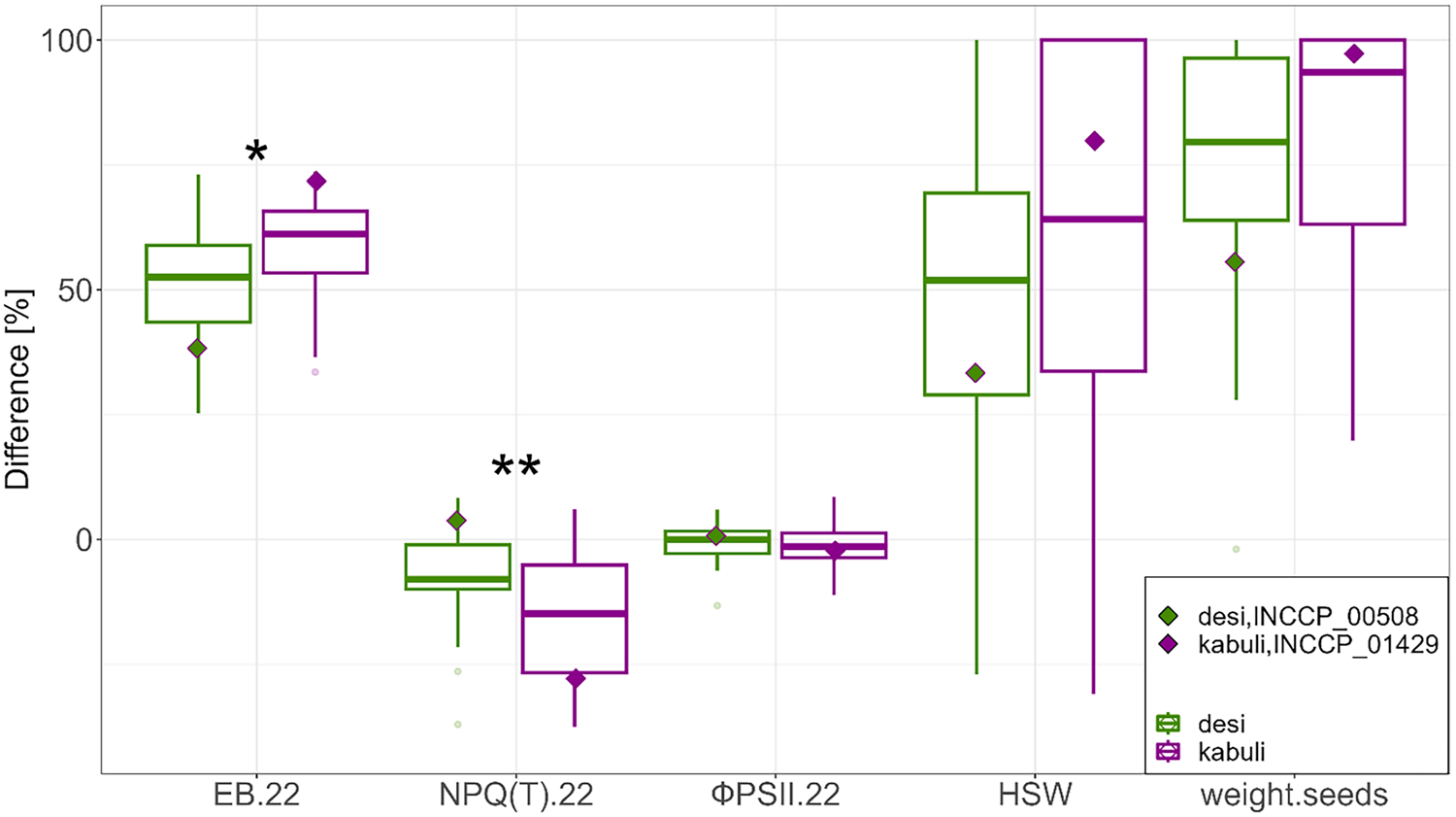
Difference of drought stress compared to control treatment of *desi* and *kabuli* for EB = Estimated Biovolume, NPQ_(T)_, ФPSII, HSW = hundred-seed-weight and weight of seeds at DAT 22. Difference [%] = (1 − (drought stress/control)) * 100. The two chickpea types *desi* and *kabuli* and one genotype of each type were highlighted. DAT 22 = days after transferring chickpeas to the High-throughput Phenotyping system with 14 days of drought stress. The two chickpea types, *desi* and *kabuli*, and one genotype of each type were highlighted. *p < 0.05; **p < 0.01. Comparisons between the two types that were not significant were not noted.

Furthermore, *kabuli* had a higher hundred-seed-weight in control treatment than *desi* (*desi* = 168.23 ± 81.7; *kabuli* = 232.25 ± 107.91), but under drought stress and for the difference of drought stress compared to the control treatment, both types did not differ significantly (Fig. 5, Tables S14, S15). For the weight of seeds, the results were similar to hundred-seed-weight. In addition, the two types did not differ in ФPSII, r2g or Mean Color Value for any DAT or between the treatments.

## Discussion

By using High-throughput Phenotyping (HTP), the interactions between genotype and environment can be precisely analyzed and specific traits can be dissected for plant breeding. In the present study, NPQ_(T)_, a trait for NPQ for High-throughput Phenotyping, has been tested for its suitability. NPQ_(T)_ could be applied to test plant genetic resources of chickpea under well-watered and drought stress conditions and be related to other traits such as EB. High repeatabilities were achieved in phenotyping using chlorophyll fluorescence and RGB imaging, which underlie the suitability of NPQ_(T)_ in pre-breeding.

The HTP system used in this study has already proven successful in the investigation of chickpea plant genetic resources under drought stress and has provided the phenotypic data basis for other studies on bread and durum wheat (*Triticum aestivum* L., *T. durum* L.) and barley to decipher the genetic architecture of drought stress by GWAS^31,32^.

For the method-validation of the experiment, the chlorophyll fluorescence measurements of F_v_/F_m_, NPQ and NPQ_(T)_ were successfully performed and showed a satisfactory and solid repeatability. An exceptionally high correlation coefficient between NPQ and NPQ_(T)_ of *r* = 0.9 illustrated that NPQ_(T)_ could be used as a valid proxy for NPQ. In addition, this result, measured with chickpea on our HTP system, was consistent with the measurements obtained during the development of the protocol of NPQ_(T)_ with *Arabidopsis*^26^.

Chlorophyll fluorescence measurements for HTP must be precise and performed quickly in order to fit into the high-throughput process. For an NPQ measurement, a dark adaptation of at least 30 min is necessary, followed by a measurement of at least 15 min^23^. Including all technically necessary transport and circulation steps, we were able to measure at least 4.1 times more single plants on our HTP system during the day with the protocol for NPQ_(T)_ than with the usual NPQ protocol during the night when all plants are dark-adapted. If NPQ is measured during the day and dark adaptation is necessary first, our NPQ_(T)_ protocol is about 40 times faster. This makes NPQ_(T)_ extremely suitable and advantageous for HTP.

For the method-implementation, during the drought stress, the repeatabilities for the image-derived traits were useful and comparable to the repeatabilities previously measured on this HTP system^32^. In a previous study, a high correlation of *r* = 0.96 was already calculated between Estimated Biovolume and dry weight of chickpea plants, so Estimated Biovolume can be used as a valid proxy for biomass^8^.

The following applications under fully light adapted conditions of NPQ_(T)_ and ФPSII for the method-implementation during drought stress and recovery were comparable in terms of repeatability with previous studies on chickpea on ФPSII^8^. Furthermore, the values of NPQ_(T)_ were similar to experiments with maize under drought stress and soybean under cold stress^28,29,33^. In addition, NPQ_(T)_was able to detect more variation within the plant genetic resources than other chlorophyll fluorescence measurements.

Drought stress has a significant negative impact on plant growth and development^34^. For the image derived traits Estimated Biovolume, plant height and Mean Color Value, there was a significant difference after 8 to 11 days of drought stress between the two treatments. This impact of drought stress was in line with previous results from chickpea and emphasized that the measurements of NPQ_(T)_ and ФPSII were made on drought-stressed chickpeas or in the recovery phase^8^. Besides the impact on Estimated Biovolume, plant height and Mean Color Value, drought stress significantly affected NPQ_(T)_ and several yield traits such as hundred-seed-weight and seed weight.

Furthermore, the high correlation between hundred-seed-weight or seed weight and Estimated Biovolume throughout the study and especially on the last imaging day DAT 37 highlighted that Estimated Biovolume was a yield- relevant trait suitable to be implemented in pre-breeding. A comparable correlation was already observed in another HTP study with different chickpea genotypes^35^.

In our study, Mean Color Value, which correlates with the chlorophyll content of various species such as *Arabidopsis*, tobacco and grapevine (*Vitis vinifera*), gave the impression of a darker green color of chickpeas under drought stress in line with our previous study and others^8,36–38^. The impression of green leaves under drought stress could be explained by a difference in the ratio of chlorophyll content and relative water, as has been investigated for chickpeas^39^. In addition, the color of the leaves could be explained by the formation of anthocyanin under stress conditions. Anthocyanins, which have a red to purple or blue color spectrum, are known to have protective properties under various stress conditions and are located between the and an increase in anthocyanin content has already been observed in a greenhouse experiment with drought stress in chickpeas^40,41^.

In studies with lettuce (*Lactuca sativa* L.), rice (*Oryza sativa* L.) and chickpeas, ФPSII decreased under drought stress^8,42,43^. In this experiment, ФPSII did not decrease compared to the control treatment. ФPSII is related to the light absorbed by the chlorophyll. When less light is absorbed by chlorophyll, ФPSII is higher^44,45^. If anthocyanin has formed in drought stress, as indicated by the Mean Color Value and the red to green color ratio, anthocyanin increase the absorption and this may lead to a decrease the absorption by chlorophyll and thus the ФPSII in this particular drought stress experiment did not decrease^46^.

However, a tolerant chickpea cultivar was also found to maintain a stable ФPSII under polyethylene glycol-induced drought stress, similar to our results^47^.

This has already been documented in brassica, soybean and common bean (*Phaseolus vulgaris* L.), under abiotic stress^48–50^. Furthermore, the higher ФPSII on the day of recovery of drought stress could be due to the reopening of the stomata which stimulates photosynthesis again as it has been observed in bread wheat^51^.

Under drought stress, the average for NPQ_(T)_ increased for the 60 genotypes. This was consistent with the results for NPQ from pot and field experiments with chickpea and mung bean (*Vigna radiata* L.) under drought stress^41,47,52^.

NPQ_(T)_ is a protective mechanism that releases excess energy in the form of heat to prevent photodamage^15^. Under these circumstances, the energy released by heat is no longer available for carbon assimilation. Comparing the difference of drought stress compared to the control treatment of Estimated Biovolume and NPQ_(T)_ for the 60 genotypes, it was visible that genotypes with a low difference of Estimated Biovolume, i.e. more tolerant genotypes, show a small change for NPQ_(T)_ under drought stress and a higher NPQ_(T)_ in control treatment than drought-sensitive genotypes. In comparison, genotypes that were more sensitive to drought stress had lower NPQ_(T)_ in control and tended to increase NPQ_(T)_. When NPQ_(T)_ was much higher in drought stress, the difference of drought stress compared to the control treatment for Estimated Biovolume was also higher, because the energy was not available for carbon assimilation. This was shown with the two types of chickpea. Similar results were documented in a pot experiment for chickpea based on two chickpea varieties^47^. The drought stress sensitive variety had a lower NPQ in the control than the drought stress tolerant variety, but a higher NPQ under drought stress than the tolerant variety.

In our study, we also found significant differences between the two types of chickpea *desi* and *kabuli*. Several studies in pot and field experiments showed that *desi* was more tolerant to drought stress than *kabuli*^7,9,53^. Proline is considered an osmoprotectant as it maintains the osmotic potential and thus the turgor of the leaves. In addition, free proline and sugar helps to stabilize macromolecules and prevents oxidative damage^7,53^. Higher levels of minerals in *desi* serve as cofactors in various osmoregulatory and antioxidant mechanisms and also contribute to drought stress tolerance^9^.

In addition to a significant difference for Estimated Biovolume, we also found a significant difference for NPQ_(T)_ between *desi* and *kabuli*. After recovery of drought stress, the two types no longer differed, but *desi* tended to have a lower difference of drought stress compared to the control treatment for hundred-seed-weight and seed weight than *kabuli*, although this difference was not significant. We have now shown that one reason for the better drought tolerance of *desi* could be the effective dissipation of excess excitation energy in the PSII efficiently measurable as NPQ_(T)_.

Drought tolerance is a quantitative trait that affects yield from the time, duration, and severity of the plants in physiological and developmental processes up to yield. Similar to the work of Kromdijk et al.^22^ in which the dynamic NPQ relaxation kinetics were measured in fluctuating light, the steady-state plasticity of NPQ_(T)_ in drought stress was used here to investigate its relation with yield in challenging environments. In this context, NPQ is a protective mechanism of photosynthesis whose plasticity is reflected in carbon assimilation.

The advantage of NPQ_(T)_ over NPQ is not only the measurement time. Environmental stresses can lead to an underestimation of F_m_ due to chloroplast movement or reflecting sustained qI^26^. In contrast, for NPQ_(T)_ the F_m_ is determined for unstressed plants and used for the calculation. Instead of NPQ, PSII maximum efficiency (F_v_′/F_m_′) could be measured, since there is a non-linear coincidence^16^. However, this consistency can be inaccurate at higher NPQ values, as changes in F_v_′/F_m_′ are indicative of the contribution of NPQ to F_q_′/F_m_′^16^.

The present study of chickpea plant genetic resources showed how NPQ_(T)_ can be successfully implemented for steady-state NPQ in High-throughput Phenotyping. We demonstrated this in a diverse plant genetic resources of chickpea, so the results are robust and valid for a wide range of genotypes. Significant differences in tolerance to drought stress could be identified between chickpeas, especially between the two types *desi* and *kabuli*, using NPQ_(T)_ in combination with other image-derived and yield traits. As studies in tobacco and soybean have shown, the potential of accelerating recovery from photoprotection represents potential for enormous seed yield increases of 33% and dry matter productivity of 15%^21,22^. HTP studies with drought or other challenging environments could include NPQ_(T)_ measurements to investigate the ability to restore the steady-state photoprotection of genotypes.

## Material and methods

### Plant material

Sixty selected genotypes of chickpea (*Cicer arietinum* L.) used in this study have been previously investigated in Lauterberg et al.^8^ (Table S1). These 60 genotypes are selected from the T-CORE collection developed in INCREASE (Intelligent Collection of Food Legumes Genetic Resources for European Agrofood Systems)^54,55^ and EMCAP (European and Mediterranean Chickpea Association Panel) projects^56^. Our set of analysed chickpea genotypes consisted of 30 *desi* and 30 *kabuli*.

### High-throughput phenotyping (HTP) experiment

We employed the experimental setup of Lauterberg et al.^8^ with modifications. The HTP system (LemnaTec-Scanalyzer 3D) used in the present study is installed in an environmentally controlled greenhouse at IPK Gatersleben (51°4902300 N, 11°1701300 E, altitude 112 m). In this HTP system, individual plants are analysed and transported in a carrier by conveyor belts to the imaging chambers. The imaging chambers are equipped with cameras for top and side view, respective for visual (Red, Green, Blue, RGB) and fluorescence imaging and a lifter which allows imaging from three different angles in side view. The balance-based watering station enabled controlled irrigation and thus defined drought stress settings.

The plant material was tested with two biological replicates per genotype and treatment. The sowing date of the experiment was the 7th of September and the last image was taken on the 28th of October 2022 on the HTP system (Table S17). Two seeds were sown in the pots and thinned out to one seedling per pot. Each pot (18.5 cm × 14.9 cm diameter) was filled with Substrate No.2 (Klasmann-Deilmann GmbH, Geeste, Germany). Plant establishment was performed for 14 days under greenhouse conditions at 24 °C day/20 °C night and a relative humidity of 67% day and 76% night. A daylight period of greenhouse lights of 15 h (from 6 am to 9 pm) and manual watering. The light intensity was 200 μmol photons m^−2^ s^−^, and controlled with shading in the greenhouse or additional assimilation light. Fourteen days after sowing plants were transferred to the HTP system run at comparable growing conditions and treatment and imaging started on 15 days after sowing. During the transfer, 7 g of fertilizer with composition of 19% total nitrogen, 9% P_2_O_5_ and 10% K_2_O was added and to each pot. In addition, a plant supporter was placed on each pot and each pot was placed into a tray so that the water was completely available for the plant. During the experiment, LemnaTec software was used to randomize the arrangement of the plants twice a week resulting in a fully randomized design. For the establishment phase of the first eight days plants were kept on a level of 65% plant available water. The plants for the control treatment were kept at this level throughout the experiment. For drought stress treatment, the irrigation level was successively lowered to 10% by withholding water (Tables S17, S7). From DAT 29 on, gradual re-watering took place, followed by irrigation to 65% plant available water content on DAT 30. Irrigation took place in two steps to allow plants to absorb all of the water. Information on daily watering based on weight before and after watering can be extracted with the HTP system software. The watering regime and simulation of drought stress were developed on this HTP system initially for barley and transferred to chickpea^8,31^.

The imaging was on a daily basis and after the last imaging at DAT 37, the plants were moved from the HTP system to a regular greenhouse for the phase of maturation until harvest and to record yield traits. The number of pods, the number of empty pods and the number of seeds were scored manually for each pot with the individual plant. For quantification of weight of seeds and hundred-seed-weight MARViN, a machine for seed analysis (MARViNTECH, Wittenburg, Germany), was used. The difference of drought stress compared to the control treatment was calculated for the yield traits.

1 $$Difference\, of\, Trait \left[\%\right]=\left( 1-\frac{{trait}_{drought\, stress}}{{trait}_{control}}\right)\times 100$$

### Image-derived traits

The images were analysed using the IAP version 2.3.0 (IAP;^57^). The traits used in this study include Estimated Biovolume ([voxel]), plant height (PH; [mm]) and Mean Color Value ([hue]). The Estimated Biovolume was calculated from the images of the top view camera and the images of three side views:

2 $$Estimated\, Biovolume \left[voxel\right]= \sqrt{{average\, pixel \,side\, area}^{2}*top\, area}$$

The plant height was based on side view imaging and the Mean Color Value on top view imaging. Mean Color Value referred to the HSV color space [hue] and provided insights in the composition of the detected color of the plant (Klukas et al., 2014). An Mean Color Value of 0.23 corresponded to a green plant, based on this model. The red to green color ratio indicated the proportion of red plant pixels divided by the number of green pixels in the HSV color space and was based on side view imaging^57^. The difference of drought stress compared to the control treatment was calculated using the above Eq. 1.

### Chlorophyll fluorescence traits

As described in Lauterberg et al.^8^, the HTP system was supplemented with a chlorophyll fluorescence camera (FluorCam; version 7) from Photon Systems Instruments (PSI; Brno, Czech Republic) to measure photosynthetic performance from the top view. The FluorCam data was analyzed using the manufacturer’s software Plant Data Analyzer (version 3). When chlorophyll fluorescence measurements were performed, daily RGB imaging was performed before or after the chlorophyll fluorescence measurements.

A detailed timeline of the experiment is given in Table S17.

For the first part of the experiment, the method validation, the maximum quantum yield of photosystem II (F_v_/F_m_) was measured during the night from DAT 4 to 5 on plants that were adapted to darkness for at least 1 h (Table S17). Therefore, minimal fluorescence level (F_0_) was determined by the application of a weak, pulsed measuring light (PAR ≤ 0.2 µmol s^−1^ m^−2^), followed by a saturating light pulse (800 ms; PAR: 4000 µmol s^−1^ m^−2^) to induce maximal fluorescence level (F_m_), as described by Tschiersch et al.^14^.

In the night from DAT 5 to 6, an experiment was carried out as a method-validation to demonstrate the correlation between NPQ and NPQ_(T)_. NPQ was calculated based on the following equation:

3 $$NPQ = (\frac{F_{m}} {F_{m}^{\prime} }) - 1$$

NPQ_(T)_ was calculated based on Tietz et al.^26^. In Tietz et al. (2017) the equation includes the assumption F_v_/F_m_ = 0.83^24^.

4 $$NPQ \left( T \right) = \left( {\frac{{\left( { \frac{1}{{\left( {1 - \left( {\frac{Fv}{{Fm}}} \right)} \right)}} } \right) - 1}}{{\left( {\frac{{F_{m}^{\prime} }}{{F_{0}^{\prime} }}} \right) - 1}}} \right) - 1 = \left( {\frac{4.88}{{\left( {\frac{{F_{m}^{\prime} }}{{F_{0}^{\prime} }}} \right) - 1}}} \right) - 1$$

In the calculation of NPQ_(T)_, we have used F_v_/F_m_ = 0.856, that was previously measured in this experiment.

To measure NPQ_(T)_ a protocol with a duration of 5 min actinic illumination (PAR: 480 µmol s^−1^ m^−2^) was used. At the beginning the F_m_ and F_0_ level was measured as described above and at the end of the actinic light phase steady-state fluorescence yield, F_s_, was recorded and the sample was exposed to a saturating light pulse to measure the maximal fluorescence yield under actinic illumination F_m_′. After the subsequent exposure of far-red light for 5 s, F_0_′ was measured. Far-red light with a peak at 733 nm was applied to oxidize the plastoquinone A (Q_A_) and the plastoquinone pool.

For the second part of the experiment, the method-implementation, NPQ_(T)_ and the operating efficiency of photosystem II (ФPSII)

5 $$\Phi {\text{PSII}} = \frac{{(F_{m}^{\prime} - F_{s} )}}{{F_{m}^{\prime} }}$$

were measured for all 60 genotypes and drought stress with subsequent recovery. The plants were light acclimated in the plant adaptation tunnel for at least 5 min according to Tschiersch et al.^14^ followed by 60 s illumination (PAR 480 μmol photons m^−2^ s^−1^) after moving into the chlorophyll fluorescence imaging chamber. Finally, maximum F_m_′ was measured during 800 ms exposure to a saturating light flash (PAR: 4000 μmol photons m^−2^ s^−1^) and F_0_′ was recorded after a subsequent illumination with far-red light for 5 s.

These measurements of NPQ_(T)_ and ФPSII took place during the day with light-adapted plants on DAT 16 (8 days of drought stress), DAT 22 (14 days of drought stress), DAT 29 (first day of recovery) and DAT 37 (8 days of recovery). The difference of drought stress compared to the control treatment was calculated using the above formula.

### Statistics

The Estimated Biovolume has been downscaled by a factor of 10^−5^ and values for red to green color ratio greater than 1.25 were removed. For statistical analysis, R studio version 4.1.2 was used. For the interpolation of the image-derived traits the package “zoo” and the spline interpolation were used. The package “ASRemL” was used to calculate the outlier within the experiment, the repeatability, and the best linear unbiased estimators (BLUEs) within the experiment. We use the following model to remove outliers and use the same model to estimate BLUEs across environments:

6 $$Trait \sim Genotype +Rep+residual,$$

while “$Genotype$” is the effect of genotype, “$Rep$” is the effect of biological replicates. For BLUE estimation and outlier detection, the genotype is set as a fixed effect, and the rest are all random effects. The outlier detection test was performed following the method M4 as described by Bernal-Vasquez et al.^58^, where the standardized residuals are used in combination with the Bonferroni-Holm test to identify an outlier.

Furthermore, the repeatability has been calculated use the same model but set genotype also as random:

7 $$Repeatability= \frac{{\sigma }_{G}^{2}}{{\sigma }_{G}^{2}+\frac{{\sigma }_{e}^{2}}{{N}_{Rep}}}$$

With ${\sigma }_{G}^{2}$ is the genotypic variance, ${\sigma }_{e}^{2}$ is the error variance and ${N}_{Rep}$, number of biological replicates. All correlations were Pearson correlations and were calculated using the “rstatix” package with the associated *p*-values. The coefficient of variation has been calculated by the ratio of σ to µ. ANOVA followed by Tukey`s test was performed to calculate the significance levels for a series of measurements such as ФPSII and NPQ_(T)_.

### Supplementary Information

Supplementary Information.

## Data Availability

The datasets used and analysed during the current study available from the corresponding author on reasonable request.

## References

[1] Ahmad, F., Gaur, P. M. & Croser, J. S. Chickpea (*Cicer arietinum* L.). In *Genetic resources, chromosome engineering, and crop improvement—Grain legumes* vol. 1, 187–217 (Taylor & Francis Group, 2005).

[2] Merga B, Haji J (2019). Economic importance of chickpea: Production, value, and world trade. Cogent Food Agric..

[3] Grillakis MG (2019). Increase in severe and extreme soil moisture droughts for Europe under climate change. Sci. Total Environ..

[4] Gowda CLL, Upadhyaya HD, Sharma S, Varshney RK, Dwivedi SL (2013). Exploiting genomic resources for efficient conservation and use of chickpea, groundnut, and pigeonpea collections for crop improvement. Plant Genome.

[5] Varshney RK (2013). Fast-track introgression of "QTL-hotspot” for root traits and other drought tolerance traits in JG 11, an Elite and leading variety of chickpea. Plant Genome.

[6] van der Maesen, L. J. G. Origin, history and taxonomy of Chickpea. In *The chickpea* 11–34 (C.A.B. International, 1987).

[7] Farooq MA, Ullah A, Lee D-J, Alghamdi SS (2018). Desi chickpea genotypes tolerate drought stress better than kabuli types by modulating germination metabolism, trehalose accumulation, and carbon assimilation. Plant Physiol. Biochem..

[8] Lauterberg M, Tschiersch H, Papa R, Bitocchi E, Neumann K (2023). Engaging precision phenotyping to scrutinize vegetative drought tolerance and recovery in chickpea plant genetic resources. Plants.

[9] Medeiros JS, Nunes Da Silva M, Carvalho SMP, Santos CS, Vasconcelos MW (2023). Low water supply differentially affects the growth, yield and mineral profile of kabuli and desi chickpeas (Cicer arietinum). Ann. Appl. Biol..

[10] Purushothaman R, Upadhyaya HD, Gaur PM, Gowda CLL, Krishnamurthy L (2014). Kabuli and desi chickpeas differ in their requirement for reproductive duration. Field Crops Res..

[11] Humplík JF (2015). Automated integrative high-throughput phenotyping of plant shoots: A case study of the cold-tolerance of pea (*Pisum sativum* L.). Plant Methods.

[12] Neumann K (2017). Genetic architecture and temporal patterns of biomass accumulation in spring barley revealed by image analysis. BMC Plant Biol..

[13] Shi R, Seiler C, Knoch D, Junker A, Altmann T (2023). Integrated phenotyping of root and shoot growth dynamics in maize reveals specific interaction patterns in inbreds and hybrids and in response to drought. Front. Plant Sci..

[14] Tschiersch H, Junker A, Meyer RC, Altmann T (2017). Establishment of integrated protocols for automated high throughput kinetic chlorophyll fluorescence analyses. Plant Methods.

[15] Müller P, Li X-P, Niyogi KK (2001). Non-photochemical quenching. A response to excess light energy. Plant Physiol..

[16] Murchie EH, Lawson T (2013). Chlorophyll fluorescence analysis: A guide to good practice and understanding some new applications. J. Exp. Bot..

[17] Chen W (2022). Purple stem Brassica napus exhibits higher photosynthetic efficiency, antioxidant potential and anthocyanin biosynthesis related genes expression against drought stress. Front. Plant Sci..

[18] Punchkhon C (2022). Role of LOC_Os01g68450, containing DUF2358, in salt tolerance is mediated via adaptation of absorbed light energy dissipation. Plants.

[19] Zhu X, Ort DR, Whitmarsh J, Long SP (2004). The slow reversibility of photosystem II thermal energy dissipation on transfer from high to low light may cause large losses in carbon gain by crop canopies: A theoretical analysis. J. Exp. Bot..

[20] Retkute R (2015). Exploiting heterogeneous environments: Does photosynthetic acclimation optimize carbon gain in fluctuating light?. J. Exp. Bot..

[21] De Souza AP (2022). Soybean photosynthesis and crop yield are improved by accelerating recovery from photoprotection. Science.

[22] Kromdijk J (2016). Improving photosynthesis and crop productivity by accelerating recovery from photoprotection. Science.

[23] Baker NR (2008). Chlorophyll fluorescence: A probe of photosynthesis in vivo. Annu. Rev. Plant Biol..

[24] Björkmann O, Demming B (1987). Photon yield of 0 2 evolution and chlorophyll fluorescence characteristics at 77 K among vascular plants of diverse origins. Planta.

[25] Long SP (2022). Into the shadows and back into sunlight: Photosynthesis in fluctuating light. Annu. Rev. Plant Biol..

[26] Tietz S, Hall CC, Cruz JA, Kramer DM (2017). NPQ(T): A chlorophyll fluorescence parameter for rapid estimation and imaging of non-photochemical quenching of excitons in photosystem-II-associated antenna complexes: New, rapid probe of non-photochemical quenching. Plant Cell Environ..

[27] Diner BA (1977). Dependence of the deactivation reactions of photosystem II on the redox state of plastoquinone pool a varied under anaerobic conditions. Equilibria on the acceptor side of photosystem II. Biochim. Biophys. Acta BBA Bioenerg..

[28] Epaku GT (2021). Stay green physiological capacity of drought tolerant maize inbred lines. Afr. Crop Sci. J..

[29] Hussain MA (2023). Comparative analysis of physiological variations and genetic architecture for cold stress response in soybean germplasm. Front. Plant Sci..

[30] Kuhlgert S (2016). MultispeQ Beta: a tool for large-scale plant phenotyping connected to the open PhotosynQ network. R. Soc. Open Sci..

[31] Dhanagond S (2019). Non-invasive phenotyping reveals genomic regions involved in pre-anthesis drought tolerance and recovery in spring barley. Front. Plant Sci..

[32] Lauterberg M (2022). Precision phenotyping across the life cycle to validate and decipher drought-adaptive QTLs of wild emmer wheat (Triticum turgidum ssp. dicoccoides) introduced into elite wheat varieties. Front. Plant Sci..

[33] Shamim MJ, Kaga A, Tanaka Y, Yamatani H, Shiraiwa T (2022). Analysis of physiological variations and genetic architecture for photosynthetic capacity of Japanese Soybean Germplasm. Front. Plant Sci..

[34] Rani A (2020). Developing climate-resilient chickpea involving physiological and molecular approaches with a focus on temperature and drought stresses. Front. Plant Sci..

[35] Atieno J (2017). Exploring genetic variation for salinity tolerance in chickpea using image-based phenotyping. Sci. Rep..

[36] Faragó D, Sass L, Valkai I, Andrási N, Szabados L (2018). PlantSize offers an affordable, non-destructive method to measure plant size and color in vitro. Front. Plant Sci..

[37] Liang Y (2017). A nondestructive method to estimate the chlorophyll content of Arabidopsis seedlings. Plant Methods.

[38] Majer P, Sass L, Horváth GV, Hideg É (2010). Leaf hue measurements offer a fast, high-throughput initial screening of photosynthesis in leaves. J. Plant Physiol..

[39] Das A (2021). Transgenic chickpea (*Cicer arietinum* L.) harbouring AtDREB1a are physiologically better adapted to water deficit. BMC Plant Biol..

[40] Gould KS (2004). Nature’s swiss army knife: The diverse protective roles of Anthocyanins in leaves. J. Biomed. Biotechnol..

[41] Macar KT, Ekmekçi Y (2008). PSII photochemistry and antioxidant responses of a chickpea variety exposed to drought. Z. Für Naturforschung C.

[42] Amitrano C, Junker A, D’Agostino N, De Pascale S, De Micco V (2022). Integration of high-throughput phenotyping with anatomical traits of leaves to help understanding lettuce acclimation to a changing environment. Planta.

[43] Khan F, Upreti P, Singh R, Shukla PK, Shirke PA (2017). Physiological performance of two contrasting rice varieties under water stress. Physiol. Mol. Biol. Plants.

[44] Habash DZ, Genty B, Baker NR (1994). The consequences of chlorophyll deficiency for photosynthetic light use efficiency in a single nuclear gene mutation of cowpea. Photosynth. Res..

[45] Harbinson J, Genty B, Baker NR (1989). Relationship between the quantum efficiencies of photosystems I and 11 in Pea leaves. Plant Physiol..

[46] Gould KS, Vogelmann TC, Han T, Clearwater MJ (2002). Profiles of photosynthesis within red and green leaves of Quintinia serrata. Physiol. Plant..

[47] Saglam A, Terzi R, Demiralay M (2014). Effect of polyethylene glycol induced drought stress on photosynthesis in two chickpea genotypes with different drought tolerance. Acta Biol. Hung..

[48] Lizana C (2006). Differential adaptation of two varieties of common bean to abiotic stress. J. Exp. Bot..

[49] Mushtaq MA (2016). Comparative leaves transcriptome analysis emphasizing on accumulation of anthocyanins in Brassica: Molecular regulation and potential interaction with photosynthesis. Front. Plant Sci..

[50] Park SJ (2010). Response of leaf pigment and Chlorophyll fluorescence to light quality in soybean (Glycine max Merr. var Seoritae). Korean J. Soil Sci. Fertil..

[51] Mu Q (2022). Photosynthesis of winter wheat effectively reflected multiple physiological responses under short-term drought–rewatering conditions. J. Sci. Food Agric..

[52] Bano H, Athar H, Zafar ZU, Ogbaga CC, Ashraf M (2021). Peroxidase activity and operation of photo-protective component of NPQ play key roles in drought tolerance of mung bean [Vigna radiata (L.) Wilcziek]. Physiol. Plant..

[53] Nisa ZU (2020). A comparative metabolomic study on desi and kabuli chickpea (*Cicer arietinum* L.) genotypes under rainfed and irrigated field conditions. Sci. Rep..

[54] Bellucci E (2021). The INCREASE project: Intelligent collections of food-legume genetic resources for European agrofood systems. Plant J..

[55] Rocchetti L (2022). Towards the development, maintenance and standardized phenotypic characterization of single-seed-descent genetic resources for chickpea. Curr. Protoc..

[56] Rocchetti L (2020). The development of a European and Mediterranean Chickpea Association Panel (EMCAP). Agronomy.

[57] Klukas C, Chen D, Pape J-M (2014). Integrated analysis platform: An open-source information system for high-throughput plant phenotyping. Plant Physiol..

[58] Bernal-Vasquez A-M, Utz H-F, Piepho H-P (2016). Outlier detection methods for generalized lattices: A case study on the transition from ANOVA to REML. Theor. Appl. Genet..

