# Supplementary material for: Implementation of theoretical non-photochemical quenching (NPQ(T)) to investigate NPQ of chickpea under drought stress with High-throughput Phenotyping
Source: Sci Rep. 2024 Jun 17;14:13970. doi: 10.1038/s41598-024-63372-6 (PMC11183218; doi:10.1038/s41598-024-63372-6)

Supplementary

Table S 1: Plant material

genotype	DOI	line code UNIVPM	synonym1	synonym2	type of chickpea	country	geographic area	biological status	donor institute	NPQ NPQ$_{(T)}$
INCCP_00004	10.18730/Z8H4J	AN_Ca_0005	CIC 235	95469	kabuli	ALB	Balkanic Europe	Landrace	IPK	
INCCP_00006	10.18730/Z8HN=	AN_Ca_0009	CIC 63	44726	desi	BGR	Balkanic Europe	Breeding Material	IPK	x
INCCP_00009	10.18730/Z8FF2	AN_Ca_0015	CIC 118	44780	desi	EGY	Nord Africa	Landrace	IPK	
INCCP_00015	10.18730/Z8GJ0	AN_Ca_0024	CIC 18	44681	desi	GRC	Balkanic Europe	Landrace	IPK	
INCCP_00017	10.18730/Z8GWA	AN_Ca_0026	CIC 21	44684	desi	GRC	Balkanic Europe	Landrace	IPK	x
INCCP_00034	10.18730/Z8FG3	AN_Ca_0053	CIC 124	44786	desi	ITA	Italy	Landrace	IPK	
INCCP_00049	10.18730/Z8JNY	AN_Ca_0077	CIC 91	78711	kabuli	ITA	Italy	Landrace	IPK	
INCCP_00078	10.18730/Z8G8V	AN_Ca_0124	CIC 160	79870	kabuli	ITA	Italy	Landrace	IPK	
INCCP_00100	10.18730/Z8JJV	AN_Ca_0169	CIC 84	44747	kabuli	SVK	East Europe	Landrace	IPK	
INCCP_00102	10.18730/Z8GV9	AN_Ca_0172	CIC 203	55301	kabuli	TUN	Nord Africa	Landrace	IPK	
INCCP_00103	10.18730/Z8H5K	AN_Ca_0175	CIC 237	95476	desi	TUN	Nord Africa	Landrace	IPK	
INCCP_00119	10.18730/Z8JHT	AN_Ca_0197	CIC 8	44671	desi	TUR	Turkey	Landrace	IPK	
INCCP_00139	10.18730/ZACT4	AN_Ca_0224	USSR-05-03-BD	W6 3498	desi	TJK	Asia	Landrace	USDA	x
INCCP_00149	10.18730/ZA0ST	AN_Ca_0238	OBRAZTZOV CHIFLIK 1	W6 10046	kabuli	BGR	Balkanic Europe	Landrace	USDA	x
INCCP_00154	10.18730/ZA10~	AN_Ca_0243	BEKESCSABAI 2	W6 11071	kabuli	HUN	East Europe	Cultivar	USDA	
INCCP_00159	10.18730/ZA151	AN_Ca_0248	1204/86	W6 11077	desi	FRA	West Europe	Landrace	USDA	
INCCP_00160	10.18730/ZA162	AN_Ca_0249	1658/85	W6 11078	kabuli	ROU	Balkanic Europe	Landrace	USDA	
INCCP_00161	10.18730/ZA173	AN_Ca_0250	125/84	W6 11079	desi	HUN	East Europe	Landrace	USDA	x
INCCP_00163	10.18730/ZA195	AN_Ca_0252	417	W6 11081	desi	DEU	West Europe	Landrace	USDA	x
INCCP_00165	10.18730/ZA1B7	AN_Ca_0254	Krokhmal #2	W6 11343	kabuli	UKR	East Europe	Cultivar	USDA	
INCCP_00166	10.18730/ZA1C8	AN_Ca_0255	Krokhmal #3	W6 11344	desi	UKR	East Europe	Cultivar	USDA	
INCCP_00190	10.18730/ZA24*	AN_Ca_0287	ILC 191	W6 14954	kabuli	RUS	Asia	Landrace	USDA	x
INCCP_00193	10.18730/Z8MGF	AN_Ca_0290	NO. 12620	PI 207470	desi	AFG	Asia	Landrace	USDA	
INCCP_00216	10.18730/Z8N71	AN_Ca_0315	RUDNICKI	PI 370420	desi	MKD	Balkanic Europe	Cultivar	USDA	
INCCP_00219	10.18730/Z8NA4	AN_Ca_0318	KLISURSKI	PI 379220	desi	MKD	Balkanic Europe	Cultivar	USDA	

INCCP_00230	10.18730/Z8NNF	AN_Ca_0330	PEDROSILLANO	PI 533672	kabuli	ESP	Iberian Peninsula	Cultivar	USDA	
INCCP_00232	10.18730/Z8NQH	AN_Ca_0334	NEGRO VICOS	PI 533676	desi	ESP	Iberian Peninsula	Cultivar	USDA	
INCCP_00251	10.18730/Z8PAU	AN_Ca_0365	CALIA	PI 572508	kabuli	ITA	Italy	Cultivar	USDA	
INCCP_00291	10.18730/Z8QJ2	AN_Ca_0410	PORQUERO NEGRO	PI 577014	desi	MEX	Central America	Cultivar	USDA	
INCCP_00295	10.18730/Z8QP6	AN_Ca_0418	CM1913	PI 577022	desi	PAK	Indian continent	Cultivar	USDA	
INCCP_00298	10.18730/Z8QS9	AN_Ca_0421	CM-1	PI 577026	desi	PAK	Indian continent	Cultivar	USDA	
INCCP_00372	10.18730/Z8RFZ	AN_Ca_0501	300685-0703D	PI 595967	desi	TUR	Turkey	Landrace	USDA	
INCCP_00429	10.18730/ZA3YG	AN_Ca_0562	WKP 88-57	W6 17612	desi	PAK	Indian continent	Landrace	USDA	
INCCP_00438	10.18730/ZA2G7	AN_Ca_0571	W6 17431	W6 17431	desi	CHN	Asia	Landrace	USDA	
INCCP_00447	10.18730/ZA2SG	AN_Ca_0580	ICC 8578	W6 17447	desi	ETH	Ethiopia	Landrace	USDA	x
INCCP_00466	10.18730/Z8TX=	AN_Ca_0600	Barichhola 5	PI 596369	desi	BGD	Indian continent	Cultivar	USDA	x
INCCP_00470	10.18730/ZA46R	AN_Ca_0604	W6 19245	W6 19245	desi	UZB	Asia	Domesticated	USDA	x
INCCP_00480	10.18730/ZA4CY	AN_Ca_0614	Floreshtskiy 58/76	W6 19957	desi	MDA	Balkanic Europe	Landrace	USDA	x
INCCP_00486	10.18730/Z8V34	AN_Ca_0620	HEERA	PI 612246	desi	AUS	others	Cultivar	USDA	x
INCCP_00488	10.18730/ZA4H=	AN_Ca_0622	ILC 72 (differential)	W6 22575	kabuli	USA	USA	Landrace	USDA	x
INCCP_00508	10.18730/Z8V89	AN_Ca_0642	ILC 10766	PI 629018	desi	SYR	Middle East	Breeding Material	USDA	x
INCCP_00509	10.18730/ZA56K	AN_Ca_0643	ARM 172	W6 23903	desi	ARM	Caucasus	Domesticated	USDA	x
INCCP_00512	10.18730/Z8V9A	AN_Ca_0646	CA2969	PI 632396	kabuli	ESP	Iberian Peninsula	Breeding Material	USDA	x
INCCP_00573	10.18730/ZA703	AN_Ca_0713	ICC 14077	W6 25914	desi	IND	Indian continent	Landrace	USDA	
INCCP_00700	10.18730/ZAAYJ	AN_Ca_0844	G2503	W6 26156	kabuli	GEO	Caucasus	Landrace	USDA	
INCCP_00709	10.18730/ZAB6T	AN_Ca_0853	TJK04:11-067	W6 26223	kabuli	TJK	Asia	Landrace	USDA	
INCCP_00792	10.18730/Z8WWR	AN_Ca_0940	ILC 176	PI 670537	kabuli	MAR	Nord Africa	Landrace	USDA	
INCCP_00853	10.18730/Z8YSB	AN_Ca_1008	ILC 563	PI 670605	kabuli	LBN	Middle East	Landrace	USDA	
INCCP_00918	10.18730/Z90T2	AN_Ca_1074	ILC 1116	PI 670671	kabuli	IRN	Middle East	Landrace	USDA	x
INCCP_00955	10.18730/Z91Z2	AN_Ca_1111	ILC 1759	PI 670720	kabuli	CHL	South America	Landrace	USDA	x
INCCP_01244	10.18730/Z9B0*	AN_Ca_1408	ILC 4312	PI 671021	kabuli	ESP	Iberian Peninsula	Landrace	USDA	
INCCP_01283	10.18730/Z9C7$	AN_Ca_1447	ILC 4427	PI 671060	kabuli	UZB	Asia	Landrace	USDA	
INCCP_01396	10.18730/Z9FRU	AN_Ca_1561	ILC 4992	PI 671175	kabuli	SYR	Middle East	Landrace	USDA	

INCCP_01429	10.18730/Z9GS*	AN_Ca_1595	ILC 5558	PI 671209	kabuli	CYP	Middle East	Landrace	USDA	
INCCP_01461	10.18730/Z9HSV	AN_Ca_1628	ILC 5791	PI 671242	kabuli	TUR	Turkey	Landrace	USDA	x
INCCP_01596	10.18730/Z9P0E	AN_Ca_1767	ILC 6146	PI 671383	kabuli	PRT	Iberian Peninsula	Landrace	USDA	
INCCP_01607	10.18730/Z9PBS	AN_Ca_1778	ILC 6193	PI 671394	kabuli	DZA	Nord Africa	Landrace	USDA	
INCCP_01615	10.18730/Z9PK~	AN_Ca_1786	ILC 6264	PI 671402	kabuli	SYR	Middle East	Landrace	USDA	
INCCP_01867	10.18730/Z9YFT	AN_Ca_2041	ILC 9757	PI 671659	kabuli	KAZ	Asia	Landrace	USDA	
INCCP_01917	10.18730/ZA012	AN_Ca_2091	ILC 11968	PI 671748	kabuli	PRT	Iberian Peninsola	Landrace	USDA	x

Table S 2: Missing rate for image-derived traits

After removing outliers and before interpolation

DAT=Days after transferring to the High-throughput Phenotyping (HTP) system; EB = Estimated Biovolume [voxel]; PH = Plant Height [mm]; MCV = Mean Color Value [hue]; r2gs = red to green color ratio (side view imaging)

	**raw data**								**final data**							
	**EB**		**PH**		**MCV**		**r2gs**		**EB**		**PH**		**MCV**		**r2gs**	
**DAT**	control	stress	control	stress	control	stress	control	stress	control	stress	control	stress	control	stress	control	stress
**1**	0.00	0.00	0.00	0.00	0.00	0.00	2.50	0.83	0.00	0.00	1.67	5.00	0.00	1.67	1.68	0.00
**2**	0.00	0.00	0.00	0.00	0.00	0.00	0.83	0.00	1.67	0.00	0.00	3.33	0.00	5.00	0.00	0.00
**3**	0.00	0.00	0.00	0.00	0.00	0.00	0.83	0.00	1.67	0.00	1.67	1.67	0.00	3.33	0.00	0.00
**4**	0.00	0.00	0.00	0.00	0.00	0.00	0.83	0.00	2.50	0.00	1.67	0.00	0.00	1.67	1.68	1.67
**5**	0.00	0.00	0.00	0.00	0.00	0.00	0.83	0.00	2.50	0.00	1.67	0.00	0.00	0.00	1.68	0.00
**6**	0.00	0.00	0.00	0.00	0.00	0.00	0.83	0.00	2.50	0.00	1.67	0.00	0.00	0.00	1.68	0.00
**7**	0.00	0.00	0.00	0.00	0.00	0.00	0.00	0.00	2.50	1.67	1.67	0.00	0.00	0.00	3.36	0.00
**8**	0.00	0.00	0.00	0.00	0.00	0.00	0.00	0.00	2.50	1.67	5.00	0.00	0.00	0.00	3.36	1.67
**9**	0.00	0.00	0.00	0.00	0.00	0.00	0.00	0.00	2.50	1.67	1.67	0.00	0.00	0.00	3.36	5.00
**10**	0.00	0.00	0.00	0.00	0.00	0.00	0.00	0.00	2.50	3.33	1.67	3.33	1.67	5.00	3.36	1.67
**11**	0.00	0.00	0.00	0.00	0.00	0.00	0.00	0.00	2.50	1.67	3.33	3.33	0.00	1.67	3.36	1.67
**12**	20.00	20.83	0.00	0.00	20.00	20.83	0.00	0.00	2.50	1.67	1.67	1.67	0.00	1.67	3.36	0.00
**13**	0.00	0.00	0.00	0.00	0.00	0.00	0.00	0.00	2.50	0.00	3.33	0.00	0.00	0.00	3.36	0.00
**14**	0.00	0.00	0.00	0.00	0.00	0.00	0.00	0.00	4.17	1.67	5.00	1.67	0.00	0.00	3.36	0.00
**15**	0.00	0.00	0.00	0.00	0.00	0.00	0.00	0.00	4.17	0.00	5.00	0.00	0.00	0.00	6.72	0.00
**16**	0.00	0.00	0.00	0.00	0.00	0.00	0.00	0.00	4.17	0.00	5.00	0.00	0.00	0.00	5.04	0.00
**17**	0.00	0.00	0.00	0.00	0.00	0.00	0.00	0.00	2.50	0.00	5.00	5.00	0.00	0.00	6.72	0.00
**18**	47.50	40.83	0.00	0.00	47.50	40.83	0.00	0.00	2.50	0.00	5.00	3.33	0.00	0.00	5.04	1.67
**19**	31.67	40.00	31.67	40.00	31.67	40.00	31.67	40.00	2.50	0.00	5.00	1.67	0.00	0.00	3.36	0.00
**20**	0.00	0.00	0.00	0.00	0.00	0.00	0.00	0.00	2.50	0.00	5.00	0.00	0.00	0.00	3.36	0.00
**21**	0.00	0.00	0.00	0.00	0.00	0.00	0.00	0.00	2.50	1.67	8.33	1.67	0.00	1.67	3.36	0.00
**22**	0.00	0.00	0.00	0.00	0.00	0.00	0.00	0.00	2.50	3.33	3.33	1.67	0.00	0.00	1.68	1.67
**23**	0.00	0.00	0.00	0.00	0.00	0.00	0.00	0.00	2.50	0.00	8.33	3.33	0.00	0.00	1.68	0.00
**24**	81.67	84.17	81.67	84.17	81.67	84.17	81.67	84.17	2.50	0.00	8.33	3.33	0.00	1.67	5.04	0.00
**25**	0.00	0.00	0.00	0.00	0.00	0.00	0.00	0.00	2.50	0.00	8.33	1.67	0.00	1.67	3.36	1.67
**26**	0.00	0.00	0.00	0.00	0.00	0.00	0.00	0.00	2.50	1.67	8.33	0.00	1.67	0.00	6.72	1.67
**27**	0.00	0.00	0.00	0.00	0.00	0.00	0.00	0.00	2.50	1.67	8.33	0.00	0.00	0.00	3.36	0.00
**28**	0.00	0.00	0.00	0.00	0.00	0.00	0.00	0.00	0.83	0.00	6.67	0.00	1.67	0.00	1.68	0.00
**29**	0.00	0.00	0.00	0.00	0.00	0.00	0.00	0.00	0.83	0.00	6.67	0.00	3.33	0.00	1.68	0.00
**30**	0.00	0.00	0.00	0.00	0.00	0.00	0.00	0.00	0.83	0.00	6.67	0.00	8.33	0.00	1.68	0.00
**31**	0.00	0.00	0.00	0.00	0.00	0.00	0.00	0.00	0.83	0.00	6.67	0.00	6.67	1.67	1.68	0.00
**32**	0.00	0.00	0.00	0.00	0.00	0.00	0.00	0.00	0.83	0.00	8.33	0.00	6.67	0.00	1.68	0.00
**33**	0.00	0.00	0.00	0.00	0.00	0.00	0.00	0.00	0.83	0.00	5.00	0.00	0.00	0.00	1.68	1.67
**34**	0.00	0.00	0.00	0.00	0.00	0.00	0.00	0.00	0.83	0.00	5.00	0.00	0.00	1.67	3.36	6.67

**35**	63.33	56.67	63.33	56.67	63.33	56.67	63.33	56.67	0.83	0.00	5.00	0.00	3.33	0.00	1.68	8.33
**36**	0.00	0.00	0.00	0.00	0.00	0.00	0.00	0.00	0.83	0.00	5.00	0.00	0.00	1.67	1.68	3.33
**37**	0.00	0.00	0.00	0.00	0.00	0.00	0.00	0.00	0.83	0.00	8.33	0.00	0.00	1.67	1.68	3.33

Table S 3: missing rate per replicate

DAT=Days after transferring to the High-throughput Phenotyping (HTP) system; EB = Estimated Biovolume [voxel]; PH = Plant Height [mm]; MCV = Mean Color Value [hue]; r2gs = red to green color ratio (side view imaging)

type of data	treatment	trait	number of genotypes	average of missing rate across all DATs per replicate	std.dev of missing rate across all DATs per replicate
**raw**	**control**	**EB**	60	6.6	3.3
		**PH**	60	4.8	2.1
		**MCV**	60	6.6	3.3
		**r2gs**	60	5	2.7
	**stress**	**EB**	60	6.6	2.7
		**PH**	60	4.9	1.9
		**MCV**	60	6.6	2.7
		**r2gs**	60	4.9	2
**final**	**control**	**EB**	60	2.1	10.9
		**PH**	60	4.8	17.2
		**MCV**	60	0.9	3.2
		**r2gs**	60	2.9	9.4
	**stress**	**EB**	60	0.6	3.2
		**PH**	60	1.1	5.1
		**MCV**	60	0.9	2.3
		**r2gs**	60	1.1	3.1

Table S 4: Missing rate and ΦPSII $NPQ_{(T)}$

Final data after outlier correction. Average missing rate for the DATs.

DAT 16 = 8 days of drought stress; DAT 22 = 14 days of drought stress; DAT 29 = first day of recovery; DAT 37 = 8 days of recovery. Based on BLUEs within experiment for 60 genotypes. DAT = days after transferring to the High-throughput Phenotyping system. $NPQ_{(T)}$ = theoretical non-photochemical quenching; ϕPSII = operating efficiency of photosystem II

		control		stress	
**trait**	**DAT**	**raw data**	**final data**	**raw data**	**final data**
ϕPSII	16	20.8	20.8	17.5	17.5
	22	0.0	0.0	0.0	0.0
	29	0.0	0.0	0.0	0.0
	37	30.8	30.8	29.2	30.8
	**average**	**12.9**	**12.9**	**11.7**	**12.1**
$NPQ_{(T)}$	16	20.8	20.8	17.5	19.2
	22	0.0	0.8	0.0	0.0
	29	0.0	1.7	0.0	0.0
	37	30.8	32.5	29.2	30.8
	**average**	**12.9**	**14.0**	**11.7**	**12.5**

Table S 5: Descriptive statistics of F_v/F_m.

Based on 60 genotypes and 240 measurements

DAT=Days after transferring to the High-throughput Phenotyping (HTP) system; Fv/Fm = maximum quantum yield of photosystem II

**treatment**	**DAT**	**trait**	**min**	**q1**	**median**	**average**	**q3**	**max**	**sd**
establishment / control	4 to 5	F_v/F_m	0.852	0.854	0.856	0.856	0.857	0.861	0.002

Table S 6: Descriptive statistics of NPQ and $NPQ_{(T)}$

Based on raw data of 20 genotypes and 23 measurements, which are highlighted in Table S1.

DAT=Days after transferring to the High-throughput Phenotyping (HTP) system; NPQ = non-photochemical quenching; $NPQ_{(T)}$ = theoretical non-photochemical quenching

**treatment**	**DAT**	**trait**	**min**	**q1**	**median**	**average**	**q3**	**max**	**sd**
establishment / control	5 to 6	NPQ	0.467	0.635	0.678	**0.685**	0.732	0.832	**0.084**
		$NPQ_{(T)}$	1.507	1.773	1.835	**1.826**	1.915	2.062	**0.147**

Table S 7: Plant available water content during drought stress.

DAT=Days after transferring to the High-throughput Phenotyping (HTP) system. Calculated as average of 120 pots with each one plant.

DAT	Plant Available Water (%)
1	71.45
2	67.45
3	66.66
4	66.34
5	65.77
6	65.62
7	65.79
8	67.16
9	60.15
10	54.38
11	49.16
12	43.18
13	39.13
14	34.61
15	31.47
16	27.65
17	23.61
18	20.74
19	18.68
20	15.40
21	14.46
22	12.63
23	10.62
24	9.57
25	9.34
26	8.71
27	9.17
28	7.81
29	9.49
30	32.53
31	64.61
32	60.03
33	62.40
34	60.32
35	62.20
36	49.73
37	60.86

Table S 8: Descriptive statistics of image-derived traits.

Difference of trait [%] = (1 − (drought stress/control))*100

DAT=Days after transferring to the High-throughput Phenotyping (HTP) system; EB = Estimated Biovolume [voxel]; PH = Plant Height [mm]; MCV = Mean Color Value [hue]; r2gs = red to green color ratio (side view imaging)

DAT	trait	treatment	min	q1	median	average	q3	max	sd
**7**	EB	control	1.3	3.4	4.6	**4.7**	5.9	10.2	1.8
		stress	1.4	3.2	4.4	**4.7**	6	9.4	1.9
		difference	−6.3	5.6	4.8	**1.7**	−2.6	7.8	−2.6
	PH	control	147.3	215.7	231.4	**232.9**	249.5	324.7	35.9
		stress	142.8	206.6	233.4	**232.4**	249.5	337	37.9
		difference	3	4.2	−0.9	**0.2**	0	−3.8	−5.8
	MCV	control	0.268	0.273	0.277	**0.276**	0.279	0.287	0.004
		stress	0.269	0.273	0.276	**0.276**	0.279	0.285	0.004
		difference	−0.3	0.3	0.3	**0.2**	−0.1	0.8	−2
	r2gs	control	0.091	0.14	0.179	**0.18**	0.209	0.325	0.053
		stress	0.063	0.145	0.178	**0.195**	0.221	0.519	0.085
		difference	31.2	−3.6	0.6	**−7.9**	−5.5	−59.8	−60.8
**28**	EB	control	35.7	72.7	99.1	**99.1**	126.1	174.5	35
		stress	11.9	17.3	19.9	**20.3**	23.7	30.6	4.5
		difference	66.7	76.2	80	**79.5**	81.2	82.4	87.1
	PH	control	341.6	460	523.2	**514.9**	555.3	679.1	72.1
		stress	246.5	333.1	354.6	**365.3**	386.5	573.7	56.9
		difference	27.8	27.6	32.2	**29**	30.4	15.5	21.2
	MCV	control	0.27	0.275	0.278	**0.278**	0.28	0.286	0.004
		stress	0.281	0.294	0.296	**0.296**	0.299	0.309	0.005
		difference	−4.1	−6.9	−6.6	**−6.6**	−6.8	−8.2	−38.3
	r2gs	control	0.073	0.104	0.121	**0.125**	0.142	0.333	0.039
		stress	0.128	0.181	0.209	**0.22**	0.245	0.451	0.062
		difference	−74.1	−73.5	−72.7	**−75.9**	−72.9	−35.7	−59.4
**37**	EB	control	90	166.4	229.9	**231.5**	287.3	397.7	75.4
		stress	34.2	55.7	71.8	**72.5**	87.5	124.9	20.9
		difference	62	66.5	68.8	**68.7**	69.5	68.6	72.3
	PH	control	406.3	570.6	629.8	**615.5**	670.2	769.4	76
		stress	313.4	413.6	439.9	**449.8**	475.2	716.1	70.7
		difference	22.9	27.5	30.2	**26.9**	29.1	6.9	6.9
	MCV	control	0.264	0.272	0.273	**0.273**	0.276	0.282	0.004
		stress	0.265	0.272	0.275	**0.275**	0.277	0.284	0.004
		difference	−0.6	−0.1	−0.4	**−0.4**	−0.6	−0.7	−12.3
	r2gs	control	0.057	0.082	0.098	**0.1**	0.113	0.197	0.026
		stress	0.091	0.115	0.13	**0.138**	0.157	0.237	0.031
		difference	−60.5	−40.8	−33	**−38.8**	−39.4	−19.9	−18.6

Table S 9: Descriptive statistics of yield traits

Based on 60 genotypes and BLUEs within the experiment. HSW = hundred seed weight; nr.empty.pods = number of empty pods; nr.pods = number of pods; nr.seeds = number of seeds; weight.seeds = weight of seeds.

Difference of trait [%] = (1 − (drought stress/control))*100

trait	treatment	min	q1	median	average	q3	max	sd
HSW	control	0	125.43	219	**200.24**	272.32	402.86	**100.23**
	stress	0	0	80	**82.22**	120.66	295.5	**73.4**
	difference	NA	100	63.47	**58.94**	55.69	26.65	**26.77**
nr.empty.pods	control	0.5	2.5	4.5	**7.33**	12.63	27.5	**6.37**
	stress	0	1	3	**3.73**	5.13	15.5	**3.55**
	difference	100	60	33.33	**49.15**	59.41	43.64	**44.2**
nr.pods	control	1	9.38	19	**21.14**	30.63	56.5	**13.61**
	stress	0	1.38	4.75	**6.56**	10.13	25.5	**6.76**
	difference	100	85.33	75	**68.98**	66.94	54.87	**50.32**
nr.seeds	control	0	6	12.5	**13.51**	20.5	38	**10.05**
	stress	0	0	1.5	**4.03**	6	24	**5.25**
	difference	NA	100	88	**70.2**	70.73	36.84	**47.76**
weight.seeds	control	0	1.41	2.87	**3.32**	5.01	12.51	**2.69**
	stress	0	0	0.31	**0.75**	0.85	6.54	**1.23**
	difference	NA	100	89.27	**77.51**	82.95	47.7	**54.49**

Table S 10: p-values for yield traits.

HSW = hundred seed weight; nr.empty.pods = number of empty pods; nr.pods = number of pods; nr.seeds = number of seeds; weight.seeds = weight of seeds.
Based on 60 genotypes and BLUEs within the experiment.

	HSW	nr.empty.pods	nr.pods	nr.seeds	weight.seeds
**control vs stress**	2.70165E−11	0.000210945	1.82487E−11	2.2319E−09	6.02875E−10

Table S 11: Descriptive statistics of ϕPS II and $NPQ_{(T)}$

Based on BLUEs within the experiment and 60 genotypes.

DAT 16 = 8 days of drought stress; DAT 22 = 14 days of drought stress; DAT 29 = first day of recovery; DAT 37 = 8 days of recovery. Based on BLUEs within 60 genotypes

$NPQ_{(T)}$ = theoretical non-photochemical quenching; ϕPSII = operating efficiency of photosystem II; DAT=Days after transferring to the High-throughput Phenotyping (HTP) system

Difference of trait [%] = (1 − (drought stress/control))*100

DAT	trait	treatment	min	q1	median	average	q3	max	sd
16	$NPQ_{(T)}$	control	1.3748	1.5254	1.6072	**1.6115**	1.6856	1.8400	**0.1148**
		stress	1.3197	1.4707	1.5586	**1.5570**	1.6387	1.8800	**0.1124**
		difference	3.65	3.92	3.11	**3.11**	2.96	−2.17	**0**
	ϕPS II	control	0.4976	0.5526	0.5648	**0.5627**	0.5794	0.5986	**0.0223**
		stress	0.5306	0.5687	0.5822	**0.5799**	0.5931	0.6111	**0.0179**
		difference	-6.63	-2.90	-3.08	**-3.07**	-2.37	-2.08	**19.99**
22	$NPQ_{(T)}$	control	1.3058	1.5110	1.5551	**1.5631**	1.6262	1.7477	**0.0958**
		stress	1.3525	1.5989	1.7194	**1.7319**	1.8553	2.1836	**0.1815**
		difference	−3.05	−5.96	−10.26	**−10.90**	−14.11	−24.57	**−80.00**
	ϕPS II	control	0.4998	0.5395	0.5573	**0.5536**	0.5698	0.5947	**0.0222**
		stress	0.5159	0.5438	0.5590	**0.5581**	0.5694	0.6021	**0.0203**
		difference	−4.00	0	0	**−1.82**	0	−1.69	**0**
29	$NPQ_{(T)}$	control	1.3628	1.4730	1.5498	**1.5538**	1.6467	1.7360	**0.1017**
		stress	1.4434	1.6173	1.6548	**1.6563**	1.7124	1.8549	**0.0884**
		difference	−5.88	−10.20	−6.45	**−7.10**	−3.64	−6.32	**10**
	ϕPS II	control	0.4953	0.5283	0.5491	**0.5449**	0.5617	0.5850	**0.0223**
		stress	0.5577	0.5839	0.5923	**0.5902**	0.5989	0.6142	**0.0113**
		difference	−12.00	−9.43	−7.27	**−9.26**	−7.14	−3.39	**50**
37	$NPQ_{(T)}$	control	1.2857	1.4212	1.5463	**1.5491**	1.6566	2.1081	**0.1465**
		stress	1.3860	1.5576	1.6184	**1.6211**	1.7101	1.8875	**0.1065**
		difference	−7.75	−9.86	−4.52	**−4.52**	−3.01	10.43	**26.67**
	ϕPS II	control	0.4488	0.5260	0.5470	**0.5408**	0.5593	0.5966	**0.0304**
		stress	0.4848	0.5438	0.5589	**0.5567**	0.5696	0.6001	**0.0206**
		difference	−6.67	−1.89	−1.82	**−3.70**	−1.79	0	**33.33**

Table S 12: Two-Way ANOVA and Tukey's test $NPQ_{(T)}$ ϕPS II.

ANOVA p-value: 0< '***'

Significant p-values for Tukey's test were highlighted in red.

The combination control:control was highlighted in blue, the combination stress:control in green and stress:stress in yellow.

DAT 16 = 8 days of drought stress; DAT 22 = 14 days of drought stress; DAT 29 = first day of recovery; DAT 37 = 8 days of recovery. Based on BLUEs within 60 genotypes

$NPQ_{(T)}$ = theoretical non-photochemical quenching; ϕPSII = operating efficiency of photosystem II; DAT=Days after transferring to the High-throughput Phenotyping (HTP) system

		ϕPS II		$NPQ_{(T)}$	
		ANOVA p-value	Tukey p-value adjusted	ANOVA p-value	Tukey p-value adjusted
DAT	22-16	***	0.000	***	0.000
	29-16	***	0.486	***	0.527
	37-16	***	0.000	***	1.000
	29-22	***	0.000	***	0.040
	37-22	***	0.050	***	0.001
	37-29	***	0.000	***	0.552
treatment	stress-control	***	0.000	***	0.000
DAT:treatment	22:control-16:control	***	0.306	***	0.395
	29:control-16:control	***	0.000	***	0.184
	37:control-16:control	***	0.000	***	0.124
	16:stress-16:control	***	0.001	***	0.251
	22:stress-16:control	***	0.949	***	0.000
	29:stress-16:control	***	0.000	***	0.500
	37:stress-16:control	***	0.833	***	1.000
	29:control-22:control	***	0.341	***	1.000
	37:control-22:control	***	0.031	***	0.999
	16:stress-22:control	***	0.000	***	1.000
	22:stress-22:control	***	0.941	***	0.000
	29:stress-22:control	***	0.000	***	0.001
	37:stress-22:control	***	0.994	***	0.189
	37:control-29:control	***	0.971	***	1.000
	16:stress-29:control	***	0.000	***	1.000
	22:stress-29:control	***	0.017	***	0.000
	29:stress-29:control	***	0.000	***	0.000
	37:stress-29:control	***	0.070	***	0.072
	16:stress-37:control	***	0.000	***	1.000
	22:stress-37:control	***	0.000	***	0.000
	29:stress-37:control	***	0.000	***	0.000
	37:stress-37:control	***	0.003	***	0.045
	22:stress-16:stress	***	0.000	***	0.000
	29:stress-16:stress	***	0.153	***	0.000
	37:stress-16:stress	***	0.000	***	0.106
	29:stress-22:stress	***	0.000	***	0.017

	37:stress-22:stress	***	1.000	***	0.000
	37:stress-29:stress	***	0.000	***	0.792

Table S 13: Correlation $NPQ_{(T)}$ and ϕPS II to image-derived traits and yield traits for DAT 16, 22, 29, 37

Shown are only coefficients of correlation r with a $p<0.05$. If a trait-treatment combination is not announced, it is not significant. DAT 16 = 8 days of drought stress; DAT 22 = 14 days of drought stress; DAT 29 = first day of recovery; DAT 37 = 8 days of recovery. Based on BLUEs within 60 genotypes. $NPQ_{(T)}$ = theoretical non-photochemical quenching; ϕPSII = operating efficiency of photosystem II; DAT=Days after transferring to the High-throughput Phenotyping (HTP) system; EB = Estimated Biovolume [voxel]; PH = Plant Height [mm]; MCV = Mean Color Value [hue]; r2gs = red to green color ratio (side view imaging); HSW = hundred seed weight; nr.empty.pods = number of empty pods; nr.pods = number of pods; nr.seeds = number of seeds; weight.seeds = weight of seeds.

Difference of trait [%] = (1 − (drought stress/control))*100

DAT	trait	treatment	$NPQ_{(T)}$	ΦPSII	EB	PH	MCV	r2gs	HSW	nr.empty.pods	nr.pods	nr.seeds	weight.seeds
16	$NPQ_{(T)}$	control	1										
		stress	1										
		both	1										
		difference	1										
	ΦPSII	control		1		−0.29			0.35				0.32
		stress		1		−0.45		0.39					
		both		1		−0.39	0.29	0.35					
		difference		1								0.33	0.36
	EB	control			1	0.59		−0.39	0.61				0.37
		stress			1	0.51		−0.45	0.43	0.4	0.41		
		both			1	0.56		−0.43	0.61		0.32	0.34	0.42
		difference			1	0.66	0.29	−0.51					
	PH	control		−0.29	0.59	1		−0.62	0.29				0.27
		stress		−0.45	0.51	1		−0.59			0.27		
		both		−0.39	0.56	1	−0.23	−0.6	0.28	0.18	0.25	0.27	0.24
		difference			0.66	1		−0.31					
	MCV	control					1						
		stress					1						
		both		0.29		−0.23	1					−0.21	
		difference			0.29		1			0.26			
	r2gs	control			−0.39	−0.62		1					−0.28
		stress		0.39	−0.45	−0.59		1					
		both		0.35	−0.43	−0.6		1	−0.23	−0.2	−0.23	−0.19	−0.24
		difference			−0.51	−0.31		1					
	HSW	control		0.35	0.61	0.29			1		0.29	0.41	0.56
		stress			0.43				1	0.57	0.79	0.71	0.74
		both			0.61	0.28		−0.23	1	0.34	0.6	0.63	0.71
		difference							1		0.64	0.66	0.72
	nr.empty.pods	control								1	0.68	0.33	
		stress			0.4				0.57	1	0.73	0.4	0.43
		both				0.18		−0.2	0.34	1	0.73	0.45	0.36
		difference					0.26			1			
	nr.pods	control							0.29	0.68	1	0.8	0.65
		stress			0.41	0.27			0.79	0.73	1	0.63	0.61
		both			0.32	0.25		−0.23	0.6	0.73	1	0.83	0.75
		difference							0.64		1	0.71	0.75
	nr.seeds	control							0.41	0.33	0.8	1	0.84
		stress							0.71	0.4	0.63	1	0.91
		both			0.34	0.27	−0.21	−0.19	0.63	0.45	0.83	1	0.89

		difference		0.33					0.66		0.71	1	0.95
	weight.seeds	control		0.32	0.37	0.27		−0.28	0.56		0.65	0.84	1
		stress							0.74	0.43	0.61	0.91	1
		both			0.42	0.24		−0.24	0.71	0.36	0.75	0.89	1
		difference		0.36					0.72		0.75	0.95	1

DAT	trait	treatment	$NPQ_{(T)}$	ΦPSII	EB	PH	MCV	r2gs	HSW	nr.empty.pods	nr.pods	nr.seeds	weight.seeds
22	$NPQ_{(T)}$	control	1	0.44		−0.27							
		stress	1	−0.58	0.53		0.33						
		both	1		−0.34	−0.32	0.51	0.35	−0.26		−0.29	−0.28	−0.23
		difference	1	−0.41	−0.33	−0.31							
	ΦPSII	control	0.44	1		−0.42		0.44					
		stress	−0.58	1	−0.55	−0.36		0.28					
		both		1	−0.21	−0.39		0.34					
		difference	−0.41	1									
	EB	control			1	0.59	−0.38		0.59				0.34
		stress	0.53	−0.55	1	0.39	0.27	−0.35	0.45	0.37	0.45		
		both	−0.34	−0.21	1	0.65	−0.66	−0.47	0.7	0.21	0.48	0.49	0.57
		difference	−0.33		1	0.71	−0.28						
	PH	control	−0.27	−0.42	0.59	1	−0.37	−0.49	0.27				0.31
		stress		−0.36	0.39	1	−0.27	−0.59			0.27		
		both	−0.32	−0.39	0.65	1	−0.55	−0.64	0.44	0.27	0.43	0.44	0.45
		difference	−0.31		0.71	1				−0.29			
	MCV	control			−0.38	−0.37	1	0.27					
		stress	0.33		0.27	−0.27	1	0.28					
		both	0.51		−0.66	−0.55	1	0.54	−0.47	−0.26	−0.48	−0.5	−0.5
		difference			−0.28		1			0.26			
	r2gs	control		0.44		−0.49	0.27	1					
		stress		0.28	−0.35	−0.59	0.28	1					
		both	0.35	0.34	−0.47	−0.64	0.54	1	−0.28	−0.24	−0.35	−0.27	−0.28
		difference						1					
	HSW	control			0.59	0.27			1		0.29	0.41	0.56
		stress			0.45				1	0.57	0.79	0.71	0.74
		both	−0.26		0.7	0.44	−0.47	−0.28	1	0.34	0.6	0.63	0.71
		difference							1		0.64	0.66	0.72
	nr.empty.pods	control								1	0.68	0.33	
		stress			0.37				0.57	1	0.73	0.4	0.43
		both			0.21	0.27	−0.26	−0.24	0.34	1	0.73	0.45	0.36
		difference				−0.29	0.26			1			
	nr.pods	control							0.29	0.68	1	0.8	0.65
		stress			0.45	0.27			0.79	0.73	1	0.63	0.61
		both	−0.29		0.48	0.43	−0.48	−0.35	0.6	0.73	1	0.83	0.75
		difference							0.64		1	0.71	0.75
	nr.seeds	control							0.41	0.33	0.8	1	0.84
		stress							0.71	0.4	0.63	1	0.91
		both	−0.28		0.49	0.44	−0.5	−0.27	0.63	0.45	0.83	1	0.89
		difference							0.66		0.71	1	0.95
	weight.seeds	control			0.34	0.31			0.56		0.65	0.84	1
		stress							0.74	0.43	0.61	0.91	1
		both	−0.23		0.57	0.45	−0.5	−0.28	0.71	0.36	0.75	0.89	1
		difference							0.72		0.75	0.95	1

DAT	trait	treatment	$NPQ_{(T)}$	ΦPSII	EB	PH	MCV	r2gs	HSW	nr.empty.pods	nr.pods	nr.seeds	weight.seeds
29	$NPQ_{(T)}$	control	1	0.44		−0.28		0.27					
		stress	1	0.28	0.3								
		both	1	0.58	−0.45	−0.48	0.37	0.38	−0.26		−0.25	−0.25	−0.3
		difference	1	0.31			−0.26						
	ΦPSII	control	0.44	1		−0.32		0.32					
		stress	0.28	1									
		both	0.58	1	−0.71	−0.7	0.74	0.6	−0.36	−0.25	−0.41	−0.36	−0.33
		difference	0.31	1									
	EB	control			1	0.67		−0.51	0.59				0.4
		stress	0.3		1	0.42		−0.47	0.45	0.39	0.5	0.29	0.26
		both	−0.45	−0.71	1	0.84	−0.77	−0.71	0.7	0.24	0.53	0.53	0.62
		difference			1	0.6		−0.3					
	PH	control	−0.28	−0.32	0.67	1		−0.67	0.32				0.3
		stress			0.42	1		−0.49					
		both	−0.48	−0.7	0.84	1	−0.71	−0.77	0.55	0.29	0.52	0.51	0.54
		difference			0.6	1				−0.29			
	MCV	control					1						
		stress					1		−0.28			−0.31	
		both	0.37	0.74	−0.77	−0.71	1	0.58	−0.52	−0.33	−0.57	−0.56	−0.54
		difference	−0.26				1		−0.3				
	r2gs	control	0.27	0.32	−0.51	−0.67		1					
		stress			−0.47	−0.49		1					
		both	0.38	0.6	−0.71	−0.77	0.58	1	−0.47	−0.28	−0.45	−0.4	−0.43
		difference			−0.3			1					
	HSW	control			0.59	0.32			1		0.29	0.41	0.56
		stress			0.45		−0.28		1	0.57	0.79	0.71	0.74
		both	−0.26	−0.36	0.7	0.55	−0.52	−0.47	1	0.34	0.6	0.63	0.71
		difference					−0.3		1		0.64	0.66	0.72
	nr.empty.pods	control								1	0.68	0.33	
		stress			0.39				0.57	1	0.73	0.4	0.43
		both		−0.25	0.24	0.29	−0.33	−0.28	0.34	1	0.73	0.45	0.36
		difference				−0.29				1			
	nr.pods	control							0.29	0.68	1	0.8	0.65
		stress			0.5				0.79	0.73	1	0.63	0.61
		both	−0.25	−0.41	0.53	0.52	−0.57	−0.45	0.6	0.73	1	0.83	0.75
		difference							0.64		1	0.71	0.75
	nr.seeds	control							0.41	0.33	0.8	1	0.84
		stress			0.29		−0.31		0.71	0.4	0.63	1	0.91
		both	−0.25	−0.36	0.53	0.51	−0.56	−0.4	0.63	0.45	0.83	1	0.89
		difference							0.66		0.71	1	0.95
	weight.seeds	control			0.4	0.3			0.56		0.65	0.84	1
		stress			0.26				0.74	0.43	0.61	0.91	1
		both	−0.3	−0.33	0.62	0.54	−0.54	−0.43	0.71	0.36	0.75	0.89	1
		difference							0.72		0.75	0.95	1

DAT	trait	treatment	$NPQ_{(T)}$	ΦPSII	EB	PH	MCV	r2gs	HSW	nr.empty.pods	nr.pods	nr.seeds	weight.seeds
37	$NPQ_{(T)}$	control	1										
		stress	1										
		both	1		−0.31	−0.34		0.33	−0.19				
		difference	1										
	ΦPSII	control		1					0.36			0.28	0.33
		stress		1		−0.5		0.28					
		both		1		−0.39	0.22	0.32					
		difference		1			0.35						
	EB	control			1	0.65		−0.46	0.53				0.39
		stress			1	0.37	−0.51	−0.32	0.39	0.39	0.45		0.27
		both	−0.31		1	0.82		−0.64	0.68	0.26	0.53	0.52	0.61
		difference			1	0.56		−0.31					
	PH	control			0.65	1		−0.6	0.28				
		stress		−0.5	0.37	1	−0.33	−0.54					
		both	−0.34	−0.39	0.82	1	−0.23	−0.73	0.53	0.3	0.51	0.48	0.52
		difference			0.56	1				−0.27			
	MCV	control					1		0.35				
		stress			−0.51	−0.33	1	0.29	−0.4			−0.3	−0.36
		both		0.22		−0.23	1	0.18			−0.18	−0.21	−0.22
		difference		0.35			1					0.34	0.33
	r2gs	control			−0.46	−0.6		1					
		stress		0.28	−0.32	−0.54	0.29	1					
		both	0.33	0.32	−0.64	−0.73	0.18	1	−0.38	−0.21	−0.34	−0.3	−0.34
		difference			−0.31			1					
	HSW	control		0.36	0.53	0.28	0.35		1		0.29	0.41	0.56
		stress			0.39		−0.4		1	0.57	0.79	0.71	0.74
		both	−0.19		0.68	0.53		−0.38	1	0.34	0.6	0.63	0.71
		difference							1		0.64	0.66	0.72
	nr.empty.pods	control								1	0.68	0.33	
		stress			0.39				0.57	1	0.73	0.4	0.43
		both			0.26	0.3		−0.21	0.34	1	0.73	0.45	0.36
		difference				−0.27				1			
	nr.pods	control							0.29	0.68	1	0.8	0.65
		stress			0.45				0.79	0.73	1	0.63	0.61
		both			0.53	0.51	−0.18	−0.34	0.6	0.73	1	0.83	0.75
		difference							0.64		1	0.71	0.75
	nr.seeds	control		0.28					0.41	0.33	0.8	1	0.84
		stress					−0.3		0.71	0.4	0.63	1	0.91
		both			0.52	0.48	−0.21	−0.3	0.63	0.45	0.83	1	0.89
		difference					0.34		0.66		0.71	1	0.95
	weight.seeds	control		0.33	0.39				0.56		0.65	0.84	1
		stress			0.27		−0.36		0.74	0.43	0.61	0.91	1
		both			0.61	0.52	−0.22	−0.34	0.71	0.36	0.75	0.89	1
		difference					0.33		0.72		0.75	0.95	1

Table S 14: descriptive statistics of $NPQ_{(T)}$, ΦPSII, EB and HSW for desi and kabuli on DAT 22.

Based on BLUEs within 60 genotypes. DAT 22 = 14 days of drought stress; DAT=Days after transferring to the High-throughput Phenotyping (HTP) system; $NPQ_{(T)}$= theoretical non-photochemical quenching; ϕPSII = operating efficiency of photosystem II; EB = Estimated Biovolume [voxel]; HSW = hundred seed weight; weight.seeds = weight of seeds.

Difference of trait [%] = (1 − (drought stress/control))*100

trait	type of chickpea	treatment	min	q1	median	average	q3	max	sd
EB	desi	control	17.35	33.72	41.13	43.57	56.92	73.58	14.88
		stress	10.71	17.30	19.95	20.10	23.65	27.45	4.29
		difference	25.20	43.48	52.55	50.73	58.88	73.10	11.91
	kabuli	control	26.30	40.08	58.21	56.64	69.83	105.83	19.91
		stress	14.26	16.93	21.76	21.60	25.13	31.79	4.97
		difference	33.50	53.37	61.15	58.42	65.70	73.72	11.19
HSW	desi	control	0.00	118.65	150.69	168.23	236.69	299.93	81.70
		stress	0.00	56.56	81.88	85.93	110.94	295.50	63.93
		difference	−40.90	28.45	51.63	46.94	68.89	100.00	37.94
	kabuli	control	0.00	193.44	267.14	232.25	303.47	402.86	107.91
		stress	0.00	0.00	75.00	78.51	134.06	273.12	82.74
		difference	−30.97	33.67	64.09	59.66	100.00	100.00	38.55
$NPQ_{(T)}$	desi	control	1.43	1.53	1.60	1.59	1.65	1.75	0.08
		stress	1.35	1.58	1.71	1.70	1.80	2.10	0.18
		difference	−37.05	−9.96	−8.02	−6.75	−1.09	8.26	10.70
	kabuli	control	1.31	1.48	1.53	1.53	1.58	1.75	0.10
		stress	1.46	1.62	1.73	1.76	1.91	2.18	0.18
		difference	−37.54	−26.67	−14.91	−15.39	−5.15	6.00	12.56
ΦPSII	desi	control	0.50	0.55	0.56	0.56	0.57	0.59	0.02
		stress	0.53	0.55	0.56	0.56	0.58	0.60	0.02
		difference	−13.26	−2.83	−0.04	−0.59	1.68	5.96	3.81
	kabuli	control	0.50	0.53	0.56	0.55	0.57	0.58	0.02
		stress	0.52	0.54	0.56	0.55	0.57	0.60	0.02
		difference	−11.15	−3.69	−1.47	−1.26	1.30	8.49	4.60
weight.seeds	desi	control	0.00	1.47	2.49	3.26	4.37	9.97	2.60

		stress	0.00	0.14	0.46	0.83	0.86	5.32	1.17
		difference	-1.96	57.67	79.25	65.42	96.15	100.00	54.62
	kabuli	control	0.00	1.15	2.95	3.38	5.20	12.51	2.83
		stress	0.00	0.00	0.15	0.67	0.79	6.54	1.30
		difference	−45.53	55.62	93.41	77.59	100.00	100.00	33.11

Table S 15: p values of desi and kabuli for EB, $NPQ_{(T)}$, ΦPSII and HSW on DAT 22

BLUEs within. Desi =30 genotypes and kabuli = 30 genotypes. P values < 0.05 are displayed bold.

Based on BLUEs within 60 genotypes. DAT 22 = 14 days of drought stress; DAT=Days after transferring to the High-throughput Phenotyping (HTP) system; $NPQ_{(T)}$= theoretical non-photochemical quenching; ϕPSII = operating efficiency of photosystem II; EB = Estimated Biovolume [voxel]; HSW = hundred seed weight; weight.seeds = weight of seeds.

Difference of trait [%] = (1 − (drought stress/control))*100

	desi vs kabuli			control vs stress	
trait	control treatment	stress treatment	difference	desi	kabuli
EB	**0.0059**	0.2229	**0.0153**	**0.0000**	**0.0000**
HSW	**0.0121**	0.6989	0.2146	**0.0001**	**0.0000**
$NPQ_{(T)}$	**0.0095**	0.1951	**0.0057**	**0.0046**	**0.0000**
ΦPSII	**0.0379**	0.2177	0.5468	0.5976	0.2592

Table S 16: Descriptive statistics for selected genotypes on DAT 22 and 37 and yield data.

DAT 22 = 14 days of drought stress; DAT=Days after transferring to the High-throughput Phenotyping (HTP) system; $NPQ_{(T)}$ = theoretical non-photochemical quenching; ϕPSII = operating efficiency of photosystem II; EB = Estimated Biovolume [voxel]; HSW = hundred seed weight; weight.seeds = weight of seeds.

Difference of trait [%] = (1 − (drought stress/control))*100

		DAT 22			DAT 37			yield traits	
**genotype**	**treatment**	**NPQ(T)**	**ϕPSII**	**EB**	**NPQ(T)**	**ϕPSII**	**EB**	**HSW**	**weight.seeds**
INCCP_00508	control	1.62	0.59	17.35	1.40	0.57	90.00	75.00	0.23
(desi)	stress	1.55	0.59	10.71	1.75	0.60	38.12	50.00	0.10
	difference	3.75	0.71	38.27	−24.82	−5.40	57.64	33.33	55.56
INCCP_01429	control	1.40	0.56	50.49	1.63	0.55	222.96	346.34	5.07
(kabuli)	stress	1.79	0.58	14.27	1.72	0.57	52.36	70.00	0.14
	difference	−27.85	−2.23	71.73	−5.66	−4.14	76.51	79.79	97.24

Table S 17: Timeline of experiment

DAS=Days after sowing; DAT=Days after transferring to the High-throughput Phenotyping (HTP) system; PAW=Plant available water. RGB = red-green-blue imaging; Fv/Fm = maximum quantum yield of photosystem II; NPQ = non-photochemical quenching; $NPQ_{(T)}$ = theoretical non-photochemical quenching; ϕPSII = operating efficiency of photosystem II

DAS	DAT	Action
0		Sowing and Pre-Cultivation in Greenhouse
**14**	**0**	**Transferring to HTP system and watering to 65 % PAW**
15	1	First image with RGB camera for image-derived traits
18	4	F_v/F_m measurement (dark-adapted plants)
19	5	F_v/F_m measurement (dark-adapted plants)
		NPQ measurement (dark-adapted plants); $NPQ_{(T)}$ (light-adapted plants)
20	6	NPQ measurement (dark-adapted plants); $NPQ_{(T)}$ (light-adapted plants)
**22**	**8**	**Initiation Drought Stress: 10% PAW**
30	16	$NPQ_{(T)}$ and ϕPSII measurement (8 days of drought stress) (light-adapted plants)
36	22	$NPQ_{(T)}$ and ϕPSII measurement (14 days of drought stress) (light-adapted plants)
**43**	**29**	**First step of recovery: + 300ml**
43	29	$NPQ_{(T)}$ and ϕPSII measurement (day of recovery) (light-adapted plants)
**44**	**30**	**Second step of recovery: 65%PAW**
51	37	$NPQ_{(T)}$ and ϕPSII measurement (8 days of recovery) (light-adapted plants)
51	37	Last Imaging on HTP system

Figure S 1: Repeatability of Estimated Biovolume (EB).

DAT=Days after transferring to the High-throughput Phenotyping (HTP) system;

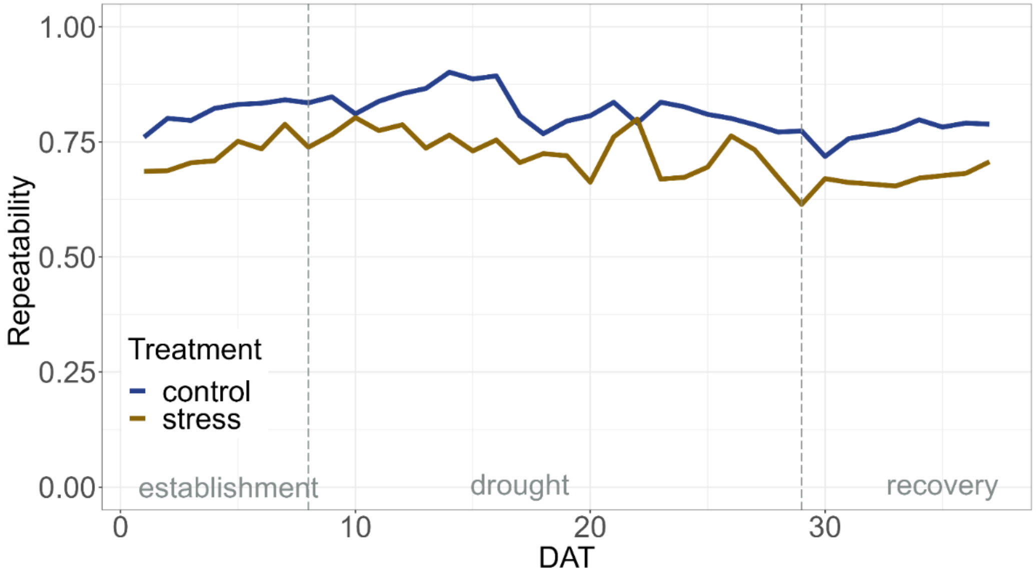

Figure S 2: Repeatability of image-derived traits.

A) PH; B) MCV; C) r2gs;

DAT=Days after transferring to the High-throughput Phenotyping (HTP) system; PH = Plant Height [mm]; MCV = Mean Color Value [hue]; r2gs = red to green color ratio (side view imaging)

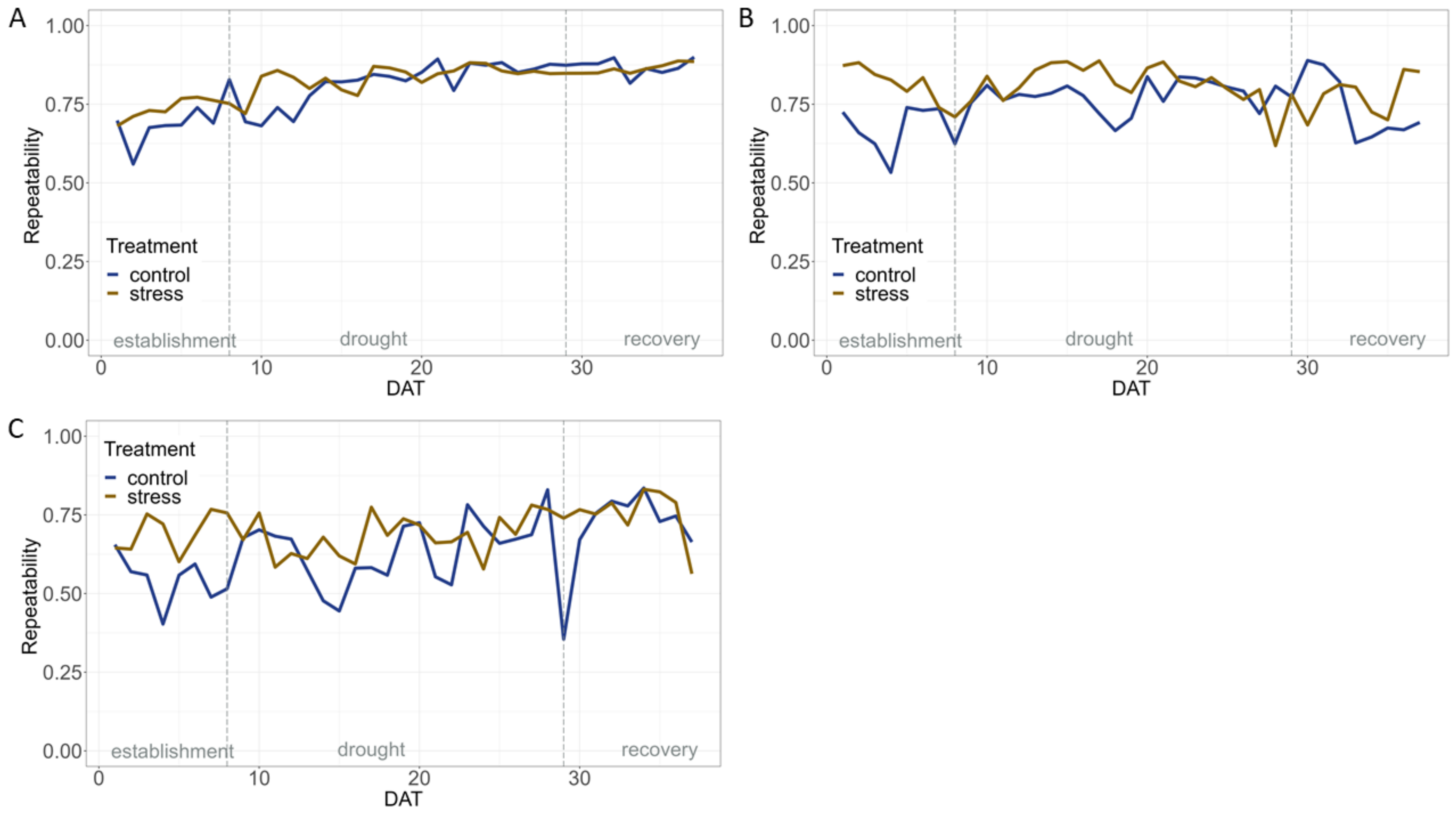

Figure S 3: Repeatability of ϕPS II and $NPQ_{(T)}$. A.) ϕPS II; B.) $NPQ_{(T)}$.

DAT 16 = 8 days of drought stress; DAT 22 = 14 days of drought stress; DAT 29 = first day of recovery; DAT 37 = 8 days of recovery. Based on BLUEs within of 60 genotypes for each control and drought stress treatment. DAT=Days after transferring to the High-throughput Phenotyping (HTP) system

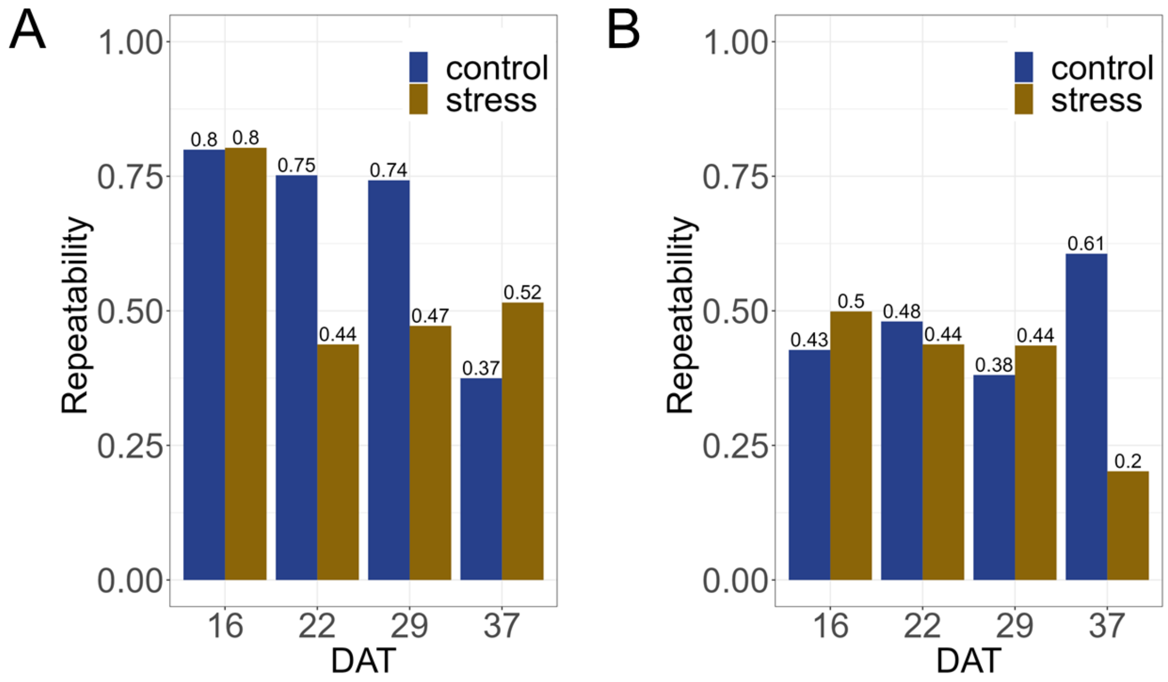

Figure S 4: Repeatability of yield traits

HSW = hundred seed weight; nr.empty.pods = number of empty pods; nr.pods = number of pods; nr.seeds = number of seeds; weight.seeds = weight of seeds.

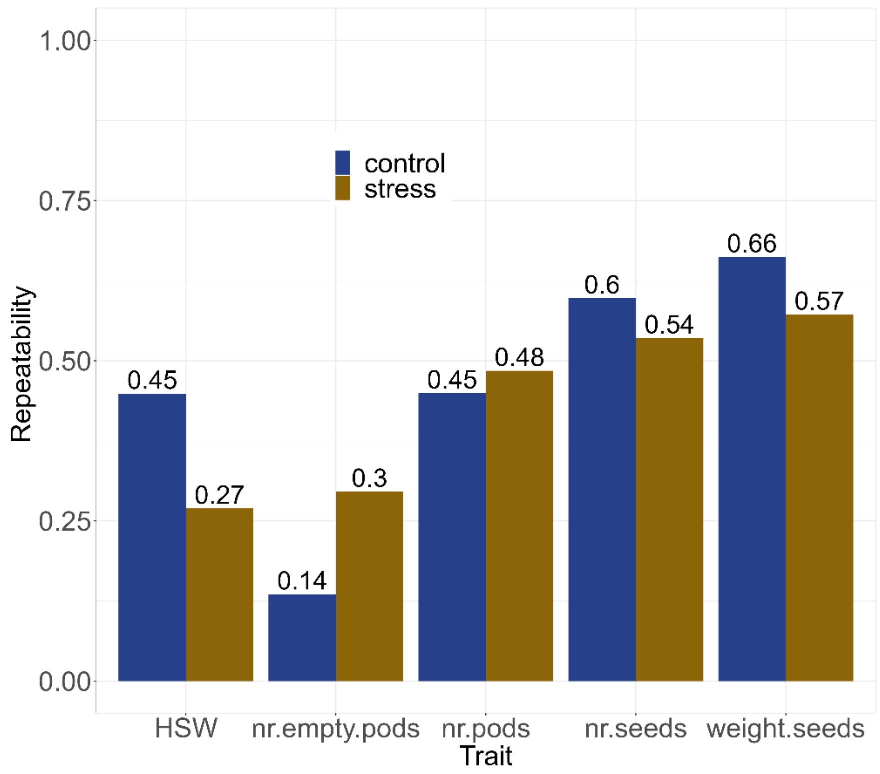

Figure S 5: Impact of drought stress on image-derived traits.

The red dashed line indicates the plant available water (PAW) to which the secondary axis refers to. The two vertical grey dashed lines indicate the different phases of the experiment: establishment, drought and recovery. The shadows describe the 95% confidence interval; as long as the shadows of the individual lines do not overlap, the significance level of $\alpha = 0.05$ was reached. Based on average of BLUEs within the experiment of all 60 genotypes. Interpolated on DAT 12, 18, 19, 24, 35. DAT = days after transferring to the High-throughput Phenotyping system.

A) Plant Height (PH); B) Mean Color Value (MCV); C) red to green color ratio, side view(r2gs)

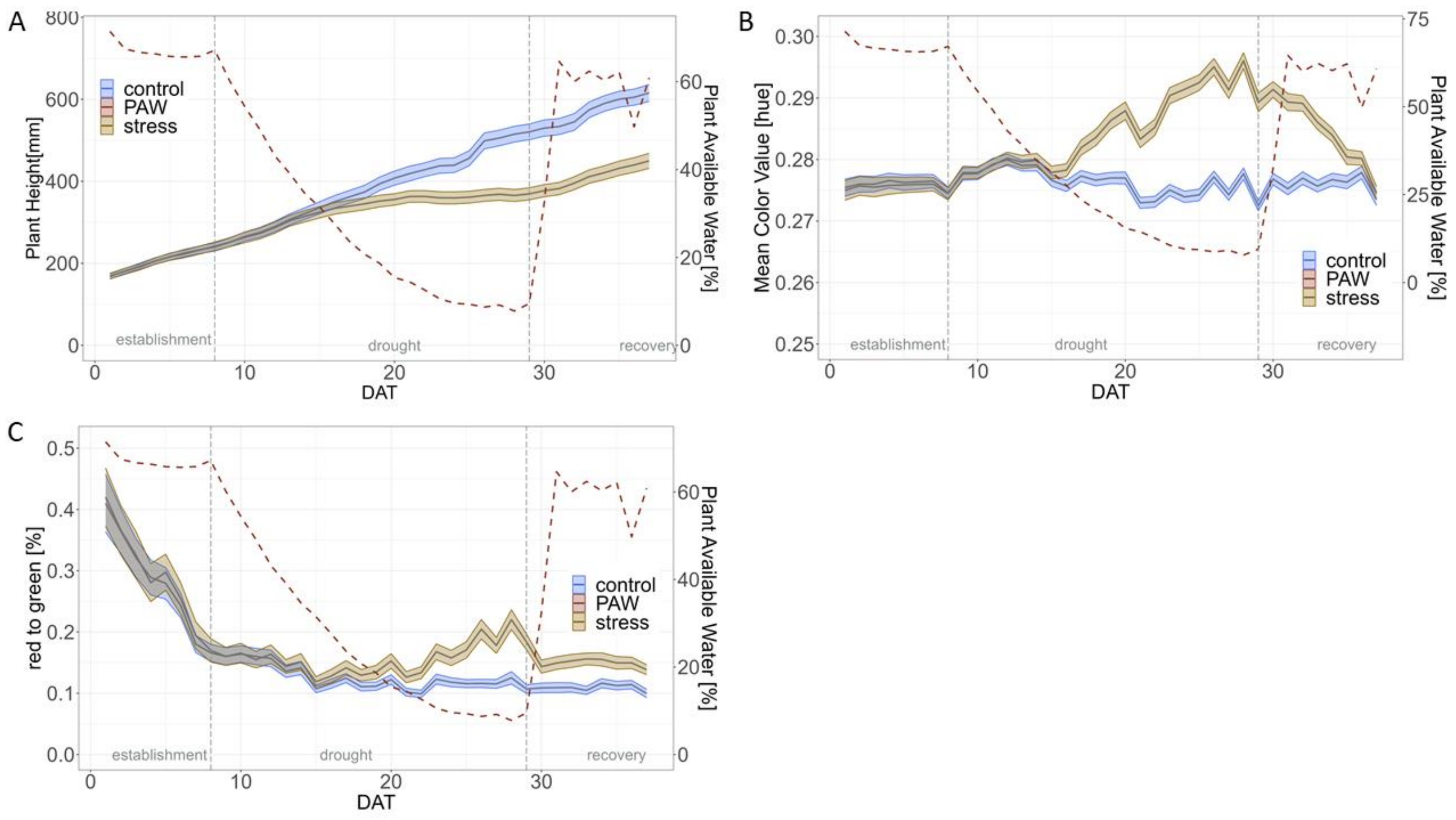

Figure S 6 Yield traits under drought stress.

Based on average of BLUEs within the experiment of all 60 genotypes. HSW = hundred-seed-weight; nr.empty.pods = number of empty pods; nr.pods = number of pods; nr.seeds = number of seeds; weight.seeds = weight of seeds.

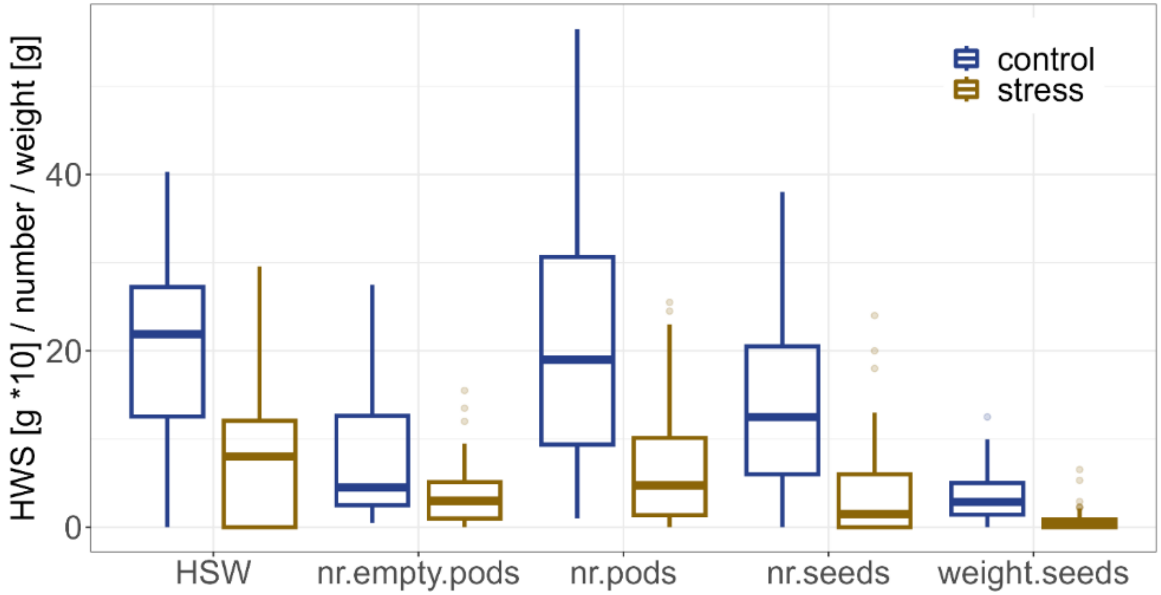

Figure S 7: Coefficient of variation of $NPQ_{(T)}$ and ϕPS II.

A.) control treatment; B.) drought stress treatment.
DAT 16 = 8 days of drought stress;
DAT 22 = 14 days of drought stress;
DAT 29 = first day of recovery;
DAT 37 = 8 days of recovery.
Based on all genotypes and BLUEs within. 60 genotypes. ϕPSII = operating efficiency of photosystem II; $NPQ_{(T)}$ = theoretical non-photochemical quenching. Coefficient of Variation [%] = (Standard Deviation / Mean) * 100; DAT=Days after transferring to the High-throughput Phenotyping HTP) system

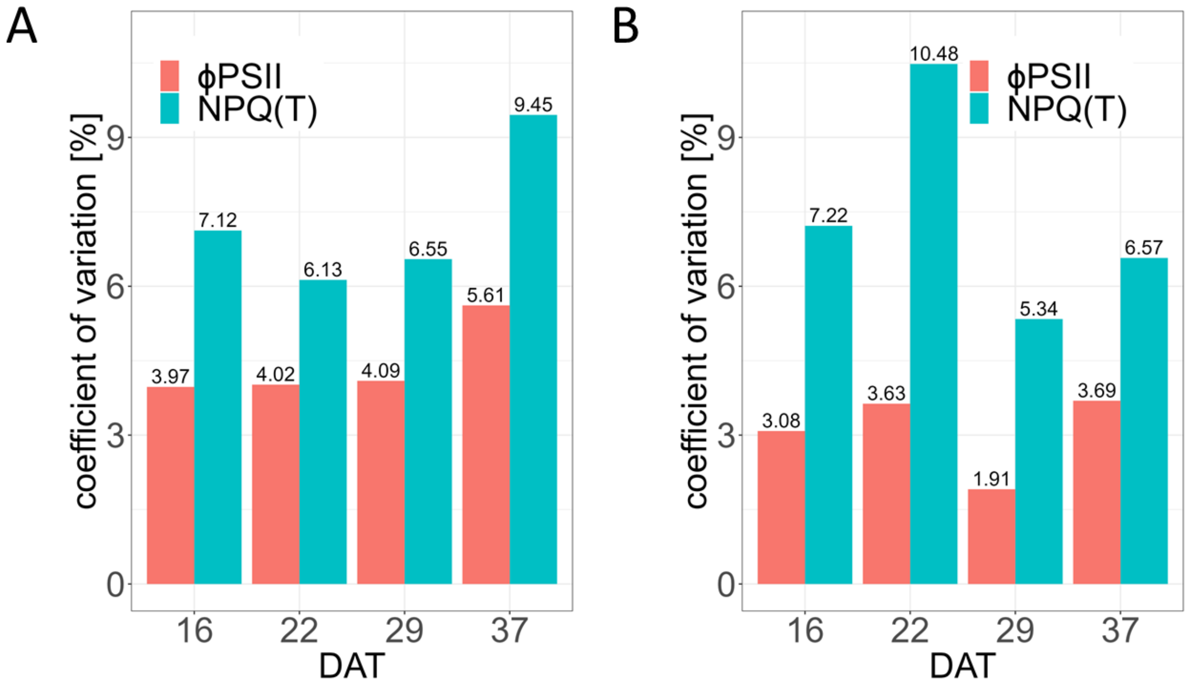

Supplement: Supplementary file 1 — Supplementary Information. [file 41598_2024_63372_MOESM1_ESM.pdf]
